# Tripartite efflux pumps of the RND superfamily: what did we learn from computational studies?

**DOI:** 10.1099/mic.0.001307

**Published:** 2023-03-27

**Authors:** Mohd Athar, Silvia Gervasoni, Andrea Catte, Andrea Basciu, Giuliano Malloci, Paolo Ruggerone, Attilio Vittorio Vargiu

**Affiliations:** ^1^​ Physics Department, University of Cagliari, Cittadella Universitaria, SP 8 km 0.700, 09042, Monserrato (CA), Italy

**Keywords:** RND efflux pumps, bacterial multi-drug resistance, antibiotics, EPIs, molecular dynamics, molecular docking

## Abstract

Bacterial resistance to antibiotics has been long recognized as a priority to address for human health. Among all micro-organisms, the so-called multi-drug resistant (MDR) bacteria, which are resistant to most, if not all drugs in our current arsenal, are particularly worrisome. The World Health Organization has prioritized the ESKAPE (* Enterococcus faecium *, *Staphylococcus aureus *, *Klebsiella pneumoniae *, *Acinetobacter baumannii *, *Pseudomonas aeruginosa* and *Enterobacter* species) pathogens, which include four Gram-negative bacterial species. In these bacteria, active extrusion of antimicrobial compounds out of the cell by means of ‘molecular guns’ known as efflux pumps is a main determinant of MDR phenotypes. The resistance-nodulation-cell division (RND) superfamily of efflux pumps connecting the inner and outer membrane in Gram-negative bacteria is crucial to the onset of MDR and virulence, as well as biofilm formation. Thus, understanding the molecular basis of the interaction of antibiotics and inhibitors with these pumps is key to the design of more effective therapeutics. With the aim to contribute to this challenge, and complement and inspire experimental research, *in silico* studies on RND efflux pumps have flourished in recent decades. Here, we review a selection of such investigations addressing the main determinants behind the polyspecificity of these pumps, the mechanisms of substrate recognition, transport and inhibition, as well as the relevance of their assembly for proper functioning, and the role of protein–lipid interactions. The journey will end with a perspective on the role of computer simulations in addressing the challenges posed by these beautifully complex machineries and in supporting the fight against the spread of MDR bacteria.

## Introduction

Bacterial efflux pumps are protein (complexes) able to expel noxious compounds, including antibiotics, out of the cell, contributing crucially to bacterial drug resistance, recognized as one of the leading public health threats in the 21st century [1, 2]. Of particular concern are the so-called multidrug pumps, which confer resistance to many, if not all, antimicrobials and are often overexpressed in clinical ‘superbug’ isolates [3–5]. Multidrug resistance (MDR) is particularly relevant for Gram-negative bacteria, which constitute the majority of the ESKAPE (* Enterococcus faecium *, *Staphylococcus aureus *, *Klebsiella pneumoniae *, *Acinetobacter baumannii *, *Pseudomonas aeruginosa* and *Enterobacter* species) pathogens prioritized by the World Health Organization [6, 7].

Among the different efflux pumps that contribute to regulating the permeability in these bacteria, a major role in resistance is played by the resistance-nodulation-cell division (RND) superfamily of secondary transporters [8–10], namely by the substrate-based subfamily known as the hydrophobe/amphiphile efflux (HAE) family [11]. The importance of RND pumps in mediating resistance and for the general physiology of bacteria is testified by the presence of several members of this family in every Gram-negative bacterium [2, 8, 10–17]. These pumps can be broadly classified into three categories (constitutively expressed, regulated and silent pumps activated by mutations in regulatory genes upon exposure to antibiotics) contributing to different physiological/pathological processes [18].

**Fig. 1.**
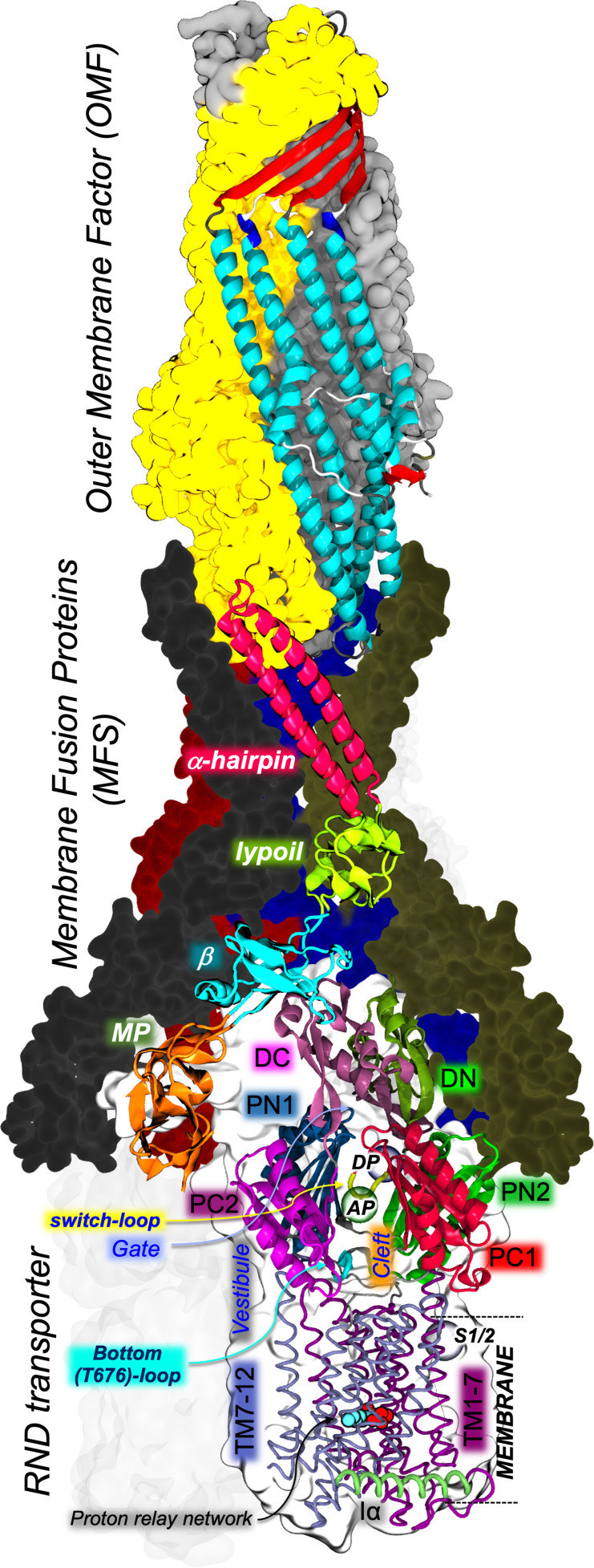
Structure of the fully assembled AcrAB-TolC efflux pump of *E. coli* as determined by cryo-electron microscopy (PDB ID: 5O66 [26]). For each component, one monomer is shown in cartoons and the remaining ones as molecular surfaces coloured differently (one AcrA monomer is transparent, while the L protomer of AcrB is hidden for the sake of clarity). AcrB and AcrA domains are indicated by black and white bold italic labels, respectively. AcrB transmembrane and periplasmic domains are shown in ribbons and cartoons, respectively. Multifunctional sites identified by X-ray crystallography (AP, DP, S1/2) are indicated by semitransparent coloured spheres and black italic labels. Additional structural elements of key relevance are indicated by coloured arrows.

The AcrAB-TolC efflux system of *Escherichia coli* (which is also the main pump in *Enterobacteriaceae* and *Salmonella Typhimurium*) and the MexAB-OprM efflux system of *Pseudomonas aeruginosa* are the paradigm models and the most well-studied RND pumps [8, 12, 14, 19]. These tripartite systems connect the inner and outer membranes of Gram-negative bacteria spanning the whole periplasm (Fig. 1) and shuttling drugs from this region out of the cell (and through a coordinated interplay with other pumps from the cytoplasm [20]). A trimeric inner membrane protein (IMP) is deputed to the substrate recognition [14, 16, 21–23] and initial transport towards a duct formed by a hexameric assembly of membrane fusion proteins (MFPs, aka perisplasmic adaptor proteins PAPs), which have a role in mediating substrate transfer through conformational coupling with the IMP [12, 24]. The MFP hexamer is linked to a trimeric outer membrane channel protein (OMP), which serves as a final duct to expel the substrate into the extracellular environment [12, 14]. Note that although the OMP and IMP proteins are homotrimers, they both bear a structural repeat and can be thus considered ‘honorary’ hexamers (indeed, six interfaces are recognizable between these two proteins) [15]. The partially redundant polyspecificities of the different RND pumps are striking properties of these proteins [15, 16], which contribute to making them a key survival tool for Gram-negative bacteria.

**Fig. 2.**
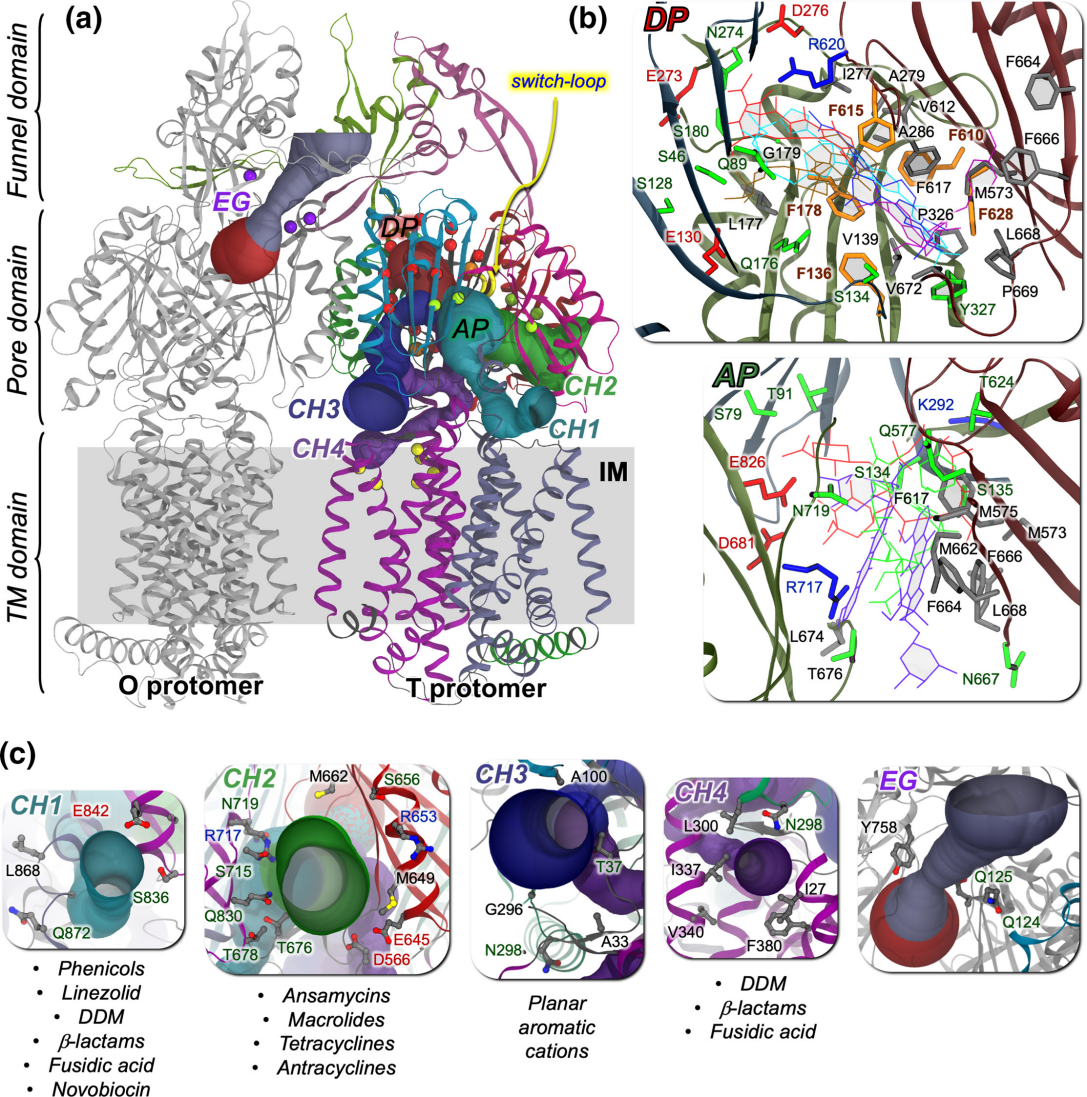
(a) Structure of the AcrB transporter of *E. coli* (PDB ID: 4D×5 [35]), with an indication of four putatively functional entry channels detected in this protein (CH1–CH4, shown as solid channels coloured differently within the T protomer; detected using CAVER [184]) and of the exit gate (EG) opening towards the Funnel Domain in the O protomer. C_α_ atoms of residues lining the multifunctional sites AP, DP (HT) and S1/2 are shown as green, red (orange) and yellow spheres, respectively. (**b**) Residues lining the DP and the AP are shown as sticks coloured by residue type (apolar, polar, negatively and positively charged residues in grey, green, red and blue, respectively). Phenylalanines lining the HT are coloured orange. Selected antibiotics and inhibitors co-crystallized within each site are also shown as sticks coloured by molecule. (c) Residues lining the entrances of transport channels CH1–4 and the bottleneck of the EG are shown as sticks coloured by atom type. Substrate specificities of each channel are reported for AcrB (taken from [30]).

Substrate extrusion is powered by the flux of protons or sodium ions through the transmembrane (hereafter TM) region of the IMP antiporter, which promotes cyclical protonation of key amino acids [12, 14–16, 23, 25]. This, in turn, induces a series of conformational changes in the periplasmic region of the IMP, which results in the opening/closing of internal channels, pushing the substrate through a peristaltic-like ‘functional rotation’ mechanism. In the simplest scheme, the IMP antiporter cycles through three distinct conformational states firstly identified in experimental structures of the transporter AcrB of *E. coli* and MexB of *P. aeruginosa *, and referred to as Loose (L; aka Access), Tight (T; aka Binding) and Open (O; aka Extrusion) [26–29]. Asymmetric conformations were indeed revealed to represent active states of the IMP antiporters, and recent work unveiled additional intermediate states of the transport cycle in AcrB [30]. The recognition of multiple, chemically unrelated compounds is believed to be mediated by:

1. The presence of two broadly specific binding sites, named access (proximal) pocket (AP) and deep (distal) pocket (DP) located between the PN1/PN2 and PC1/PC2 subdomains of the IMP transporter (Fig. 2). Multiple substrates have been co-crystallized within both the AP and DP, supporting the presence of multifunctional sites distributed within them and enabling multidrug binding [10, 12, 14–16, 21, 23, 31]. In addition, differences between the AP and DP in the relative content of aromatic, charged, and polar residues are also suggested to affect substrate preferences [16]. The DP has been dissected into two subsites, named the groove and the cave, which were proposed to mediate the binding of different substrates [32]. The bottom of the DP is lined, in AcrB, MexB and other IMPs, by a cluster of phenylalanines, known as the hydrophobic trap (hereafter HT), which binds both substrates and, more tightly, inhibitors of this transporter [16, 33, 34]. The AP and DP are separated by a switch-loop (aka G-loop, Fig. 2a), whose conformational flexibility has been shown to be key for the transport of several substrates, and it is possibly straddled by inhibitors of the RND transporter AcrB from *E. coli* [35–37].
2. The presence of multiple channels (detected in AcrB but probably present in other RND transporters as well) within IMP allow sequestering of substrates from both the periplasm and the outer leaflet of the inner membrane (IM) [10, 12, 14–16, 21, 23, 31]. The latter transport route should start at two grooves formed between TM helices 1 and 2 (TM1 and TM2) or TM7 and TM8 (hereafter CH4 and CH1, respectively) (Fig. 2a, b) [38, 39]. Recently, Pos and coworkers reported the asymmetric structure of AcrB in complex with fusidic acid bound at CH1 and CH4, and deeply embedded in the transmembrane domain, all at the same time [30]. The main route for substrates floating in the periplasm is thought to be a cleft formed by PC1/PC2 subdomains (hereafter CH2; Fig. 2a, b) [35, 40]. CH2 is open in both L and T protomers, although the PC1/PC2 cleft is wider in the former. An additional channel (hereafter CH3) was suggested to take up substrates from an inner cavity formed between the three IMP protomers [41]. CH3 seems to be the preferred entry gate for planar, cationic aromatic compounds of low molecular mass (such as ethidium). Noteworthy, CH3 bypasses the AP, and the switch-loop that separates this pocket from the DP.

Given the complexity of the RND efflux systems and of the processes they mediate, it is not surprising that, despite the advances made in recent decades, our knowledge of the molecular details regarding their functioning (which is crucial from a drug design perspective) is still limited (to give an example, the ability of RND transporters to recognize many unrelated antibiotics makes it very difficult to rationalize the effect of point mutations at the putative binding site) [1, 8, 10, 12, 15, 17, 31]. From more than a decade, several computational labs have contributed to unveiling these details using different algorithms and models [12, 16, 21, 31, 42–46]. With respect to our latest review on the subject [42], many studies on transporters other than AcrB or MexB have been published, as well as new investigations into the role of the MFPs and on the whole assembly. Here, we will provide an updated summary of most such studies, addressing the following aspects in detail:

- Molecular determinants for the polyspecificity to RND efflux pumps (including the impact of mutations on substrate recognition);
- Mechanism of substrate transport (including allosteric coupling between the transmembrane and periplasmic domains of the RND transporters, as well as the role of MFP and OMF proteins);
- Mechanisms of inhibition of the RND efflux pumps; and
- Mechanism of (and stability of the) tripartite assembly and relevance of lipid–protein interactions.

We will conclude our review with an indication of possible key topics that could benefit from computational studies, suggesting also new methodologies that can help in addressing the unsolved issues.

## Polyspecific substrate recognition in RND efflux pumps

Protein polyspecificity derives from the evolutional fitness that it brings to species, allowing them to adapt to different environments and variations of nutritional availability [47, 48]. In bacteria, this comes with several advantages, such as the enhancement of pathogenicity, better cell–cell communications, more efficient biofilm formation and greater efflux of antimicrobial compounds [2, 14, 31, 49, 50].

**Fig. 3.**
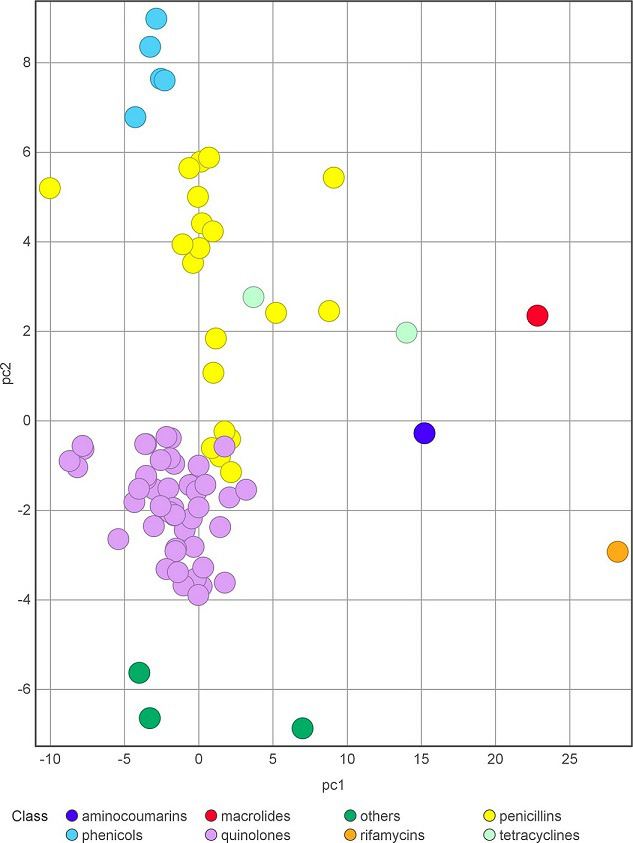
Principal component (PC) analysis of the computed physico-chemical properties of some AcrB substrates. Lists of substrates and molecular descriptors were retrieved from [12] and [57], respectively. Compounds are coloured according to the class they belong to. PC1 mainly includes size-related descriptors (e.g. molecular weight, volume), and PC2 shape-related descriptors (e.g. acylindricity, asphericity, expressing deviation from spherical and cylindrical geometry, respectively). Percentage of variance, PC1 : 45.44%; PC2: 16.92 %.

The complex structure and functional mechanism of RND transporters give them an unmatched ability to bind ligands belonging to a wide chemical space [8, 10, 12, 16, 31]. For instance, AcrB is known to bind and transport a plethora of compounds, including macrolides, fluoroquinolones, tetracyclines, chloramphenicol, doxorubicin, acriflavine, aminocoumarins, rhodamine 6G, ethidium and some β-lactams [21, 51–56]. The computed physico-chemical features of these compounds [57] cover a wide portion of the chemical space, as exemplified by principal component analysis [PCA, a mathematical technique used to analyse datasets containing a large number of entries, each associated with several dimensions (features), in order to facilitate their interpretability and visualization] reported in Fig. 3 for a series of AcrB substrate classes [12].

As mentioned in the Introduction, among the sources of such promiscuity is the presence of two multifunctional binding pockets with different amino acid compositions, yielding distinct general physical–chemical properties [21, 42, 43, 58, 59], and the existence of multiple channels promoting the uptake of different compounds [15, 39, 41, 60–62] (Fig. 2).

### Role of the main IMP binding sites for polyspecific recognition

**Fig. 4.**
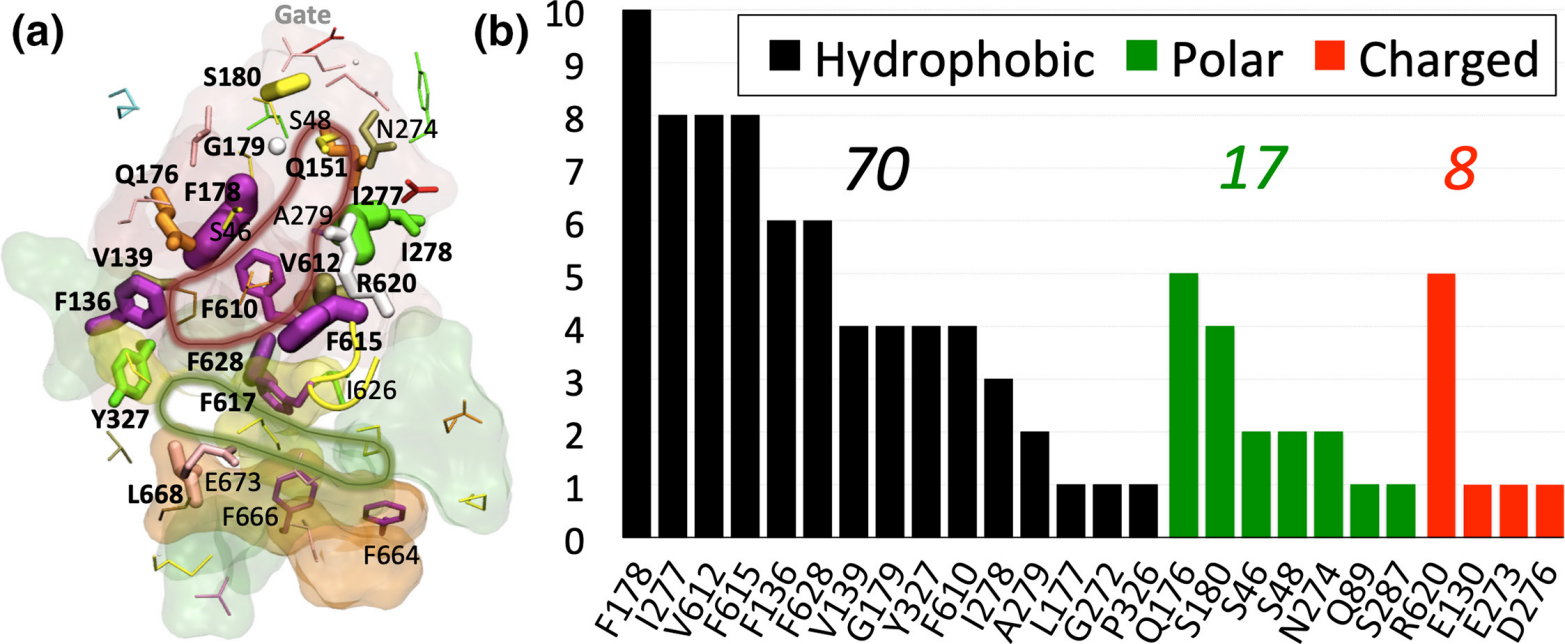
The multifunctional character of the DP of the RND transporter AcrB (adapted from [36]). (**a**) Residues contributing to the recognition of AcrB substrates are shown in sticks, with widths proportional to the frequency of binding contacts established with ten different ligands during all-atom MD simulations. The DP, AP, Cleft and AP/DP interface are shown in red, green, orange and yellow transparent surfaces, respectively, while the tip of the switch-loop is shown as a yellow tube. Bold labels refer to residues contributing to the binding of at least three substrates (inhibitors or not) of AcrB. The dark-red line highlights the contour of the DP according to this analysis. (**b**) Frequency of contribution (larger than kT – 0.593 kcal mol^–1^ – at room temperature) to the binding free energy of substrates by hydrophobic (black bars), polar (green) and charged (red) residues. The sum of all frequencies is reported above each histogram.

In this section, we will review computational studies addressing multidrug recognition in RND efflux pumps. Nikaido and co-workers employed molecular docking in the first computational investigation assessing the binding modes of several compounds (including substrates, inhibitors and non-substrates) to the DP in the T monomer of AcrB [32]. The authors found that most compounds bind either within a narrow groove at one end of the DP, or to a wide cave at the other end of the same pocket; a third group of compounds was found in between the groove and the cave. A subsequent study that combined docking, all-atom MD simulations and binding free energy calculations somewhat blurred this distinction while confirming the exceptional promiscuity of the wide DP [36]. MD simulations and free energy calculations demonstrated how this pocket exploits virtually all interaction types to stabilize the binding of different unrelated compounds. In agreement with transport experiments performed in intact cells [61], the study found that residues F136, Q176, F178, I277, V612, F615, R620 and F628 contribute to the stabilization of most substrates (Fig. 4).

The presence of two ‘multifunctional sites’ (MSs, able to bind aromatic, hydrophobic and polar groups) within the groove and cave sub-pockets was previously demonstrated by Imai *et al.* [63], who employed a fragment-based approach to perform a functional mapping on the internal surfaces of AcrB. Importantly, the same study identified different binding sites in each AcrB monomer, which is consistent with a smooth transport mechanism whereby substrates are not trapped by site-specific interactions during functional cycling of the transporter. Imai *et al.* also indicated that a subtle free-energy balance due to weakly polar and hydrophobic interactions can stabilize AcrB substrates, a result in line with [36] and with the multisite drug oscillation hypothesis proposed by Yamaguchi and co-workers to explain polyspecificity [23]. This idea was further established by a computational study employing Markov chain Monte Carlo simulations to show how the diffuse binding of solvents, acriflavine and minocycline to AcrB contributes significantly to the binding affinity [64].

The presence of AP and DP binding sites in the L and T monomers of several RND transporters from different species (in addition to AcrB from *E. coli,* MexB from *P. aeruginosa *, AdeB from *Acinetobacter baumannii *, OqxB from *Klebsiella pneumoniae *, MtrD from *Neisseria gonorrhoeae *) was confirmed by crystallographic studies [12]. In particular, the structures of AcrB in complex with different ligands revealed a preference, *viz* high molecular mass (HMM) ligands such as erythromycin preferentially bind at the AP [40, 65], while smaller low molecular mass (LMM) compounds, such as levofloxacin, doxorubicin and minocycline, bind at the DP [27, 34, 35, 66]. Importantly, the sub-sites within the large DP (i.e., cave, groove and HT) feature different hydrophobicity indexes [21, 36, 67, 68].

Ramaswamy *et al.* [43] focused on the impact of physico-chemical and topographical properties of these pockets on the promiscuity of AcrB and AcrD from *E. coli *. They found that the DP is characterized in both proteins by greater lipophilicity compared to the AP, which by contrast is constituted mainly by polar residues and is more exposed to the solvent. In addition, a higher number of MSs was detected in AcrB than in AcrD within the DP and at the interface between the two pockets, in line with the higher polyspecificity of the former transporter [12, 21]. The location of the MSs within the DP of AcrB is in good agreement with the structural data on substrate binding, and some of the MSs in AcrD are proximal to key residues for the recognition of anionic β-lactams by this protein [58]. The same authors performed an exhaustive atomic-level comparison between the two main binding pockets of MexB and MexY from *P. aeruginosa* [59], evincing in both proteins the presence of several MSs. Very recently, Catte *et al.* [69] performed atomistic MD simulations of MexB, MexY and MexF using a more realistic model of the inner phospholipid membrane and updated force-fields. The analysis of physico-chemical properties of these transporters coupled to fragment-based mapping with several organic probes revealed the presence (also in MexF) of a few MSs at locations equivalent to the AP and DP, also detected earlier in experimental structures of MexB. Moreover, it was also discovered that the channel gates deputed to the peripheral recognition of substrates are endowed with polyspecific binding abilities. Based on their findings, the authors proposed a common ‘recognition topology’ characterizing Mex transporters, which can be exploited to optimize transport and inhibition propensities of antimicrobial compounds.

Malvacio *et al.* [67] developed a computational protocol based on ensemble-docking, MD simulations and free energy calculations to rationalize the propensity of congeneric compounds (i.e., compounds bearing a similar scaffold with minor chemical modifications) as good or poor substrates of AcrB in terms of their possible binding modes to this transporter. As in several publications, good vs. poor substrates were experimentally differentiated in terms of fold differences between compound MICs in wildtype and ΔAcrB *E. coli* strains. However, since these changes could be uncorrelated with real efflux activity [70, 71], *in silico* studies were performed to better understand the possible relevance of efflux-mediated transport in resistance. The analysis of several MD trajectories, each starting from a different docking pose within the DP, indicates that poor substrates are highly stable in the HT and in the AP. Conversely, good substrates interacted marginally with the HT, and it explored multiple and energetically equivalent binding modes across the DP, all characterized by relatively high hydration and stabilized by different interactions (i.e., salt-bridges, H-bonds, and van der Waals). Furthermore, only the binding of the good substrate induced a displacement of TM8, leading to an intermediate conformation between the T and O states. This last finding provided further evidence that major conformational changes can affect the polyspecificity of the transporter (see below). Along the same lines, Atzori *et al.* [72] investigated the molecular basis for the good and poor interaction of two cephalosporins, cefepime and ceftazidime, with AcrB. They found that cefepime makes relevant although not unique interactions with the HT, as opposed to ceftazidime, which binds preferentially outside that region. The data supported surface complementarity between a compound and the RND transporter, rather than the intrinsic hydrophobicity of the former, as a key feature triggering the allosteric changes needed to transport substrates. In a second study, the same authors employed computer simulations and experiments to rationalize how differences in the physico-chemical properties of the similar carbapenem compounds, namely imipenem and meropenem, do affect their interactions with AcrB [73]. MD simulations and free energy calculations indicated that meropenem has a higher affinity than imipenem towards the DP, and both carbapenems have similar affinities towards the AP. The results are consistent with titration experiments, which indicate a low but clear affinity to AcrB only for meropenem.

Collu *et al.* [74] used a similar protocol to perform the first study providing a molecular rationale for the experimental evidence indicating that meropenem and imipenem are respectively a good and poor substrate of MexB from *P. aeruginosa *. Interestingly, the binding affinity of meropenem to the DP is higher than that of imipenem, in agreement with experimental findings [75] and other computational studies [76], and both compounds poorly interacted with the AP, confirming the role of these pockets in recognizing different types of compounds. Further analyses revealed that these differences depended also on the interactions between the ligands and the solvent (with imipenem and not meropenem being able to establish long-lasting water-mediated interactions with the DP), which is another important feature of polyspecificity (see below).

Overall, these studies are consistent with the experimental binding modes of substrates such as doxorubicin [35], minocycline [34, 40], rhodamine 6G [34], doxycycline, fusidic acid, levofloxacin [66] and puromycin [26], all interacting with some residues of the HT of AcrB. We suggested that the good and poor substrates of AcrB (and other IMP antiporters) could bind to this site with different strengths and thus have different dwelling times in that region of the protein [16, 77]. Essentially, good substrates are expected to have a binding affinity to this site that is high enough to promote allosteric structural changes in the TM region of the IMP, but low enough to allow detachment of the substrate in response to the feedback conformational change induced by the proton flux across the TM region.

A subsequent study from Dey *et al.* [78] combined evolutionary sequence analyses and molecular docking to rationalize the known antibiotic substrate selectivity differences between the RND transporters MexY and MexB of *P. aeruginosa *. The authors reported that different classes of antibiotics can bind with similar affinity to the DP of the two proteins (despite differences between the two isoforms), and concluded that to be expelled efficiently, antibiotic substrates must possess a ‘Goldilocks affinity’: binding strong enough to allow interaction with the transporter but not so tight as to impede movement through the pump.

Like AcrB and MexB, also the DP of the transporter OqxB in *K. pneumoniae* is mainly hydrophobic, despite the presence of three negatively (E50, D87 and E184) and three positively (R48, R157 and R774) charged residues (absent in other transporters) [79]. Nonetheless, OqxB can bind various molecules such as *N*-dodecyl-β-d-maltoside (DDM) and fluoroquinolone antibiotics, as revealed by both experimental [80] and computational [79] analyses. In the latter reference, docking and MD simulations revealed that the same residues that participate in H-bond interactions with the hydroxyl groups of the DDM terminal sugar ring (R48 and R157) are those interacting with the zwitterionic fluoroquinolones, together with E50, and are crucial for the optimal binding orientation of antibiotics. As seen in other studies [34, 81, 82], the solvent has a key role, with up to five structured water molecules found to mediate H-bonding networks.

Chitsaz *et al.* employed molecular docking and MD simulations to investigate the structural determinants of substrate interaction with the MtrD transporter from *N. gonorrhoeae* (homologous to AcrB from *E. coli *) [83]. They demonstrated that the cleft located between PC1 and PC2 domains of the protein is a possible entry gate for substrates, and by means of *in silico* mutagenesis calculations, they also identified several residues located within the AP, DP and HT sites and involved in non-specific binding of MtrD substrates.

Vergalli *et al.* [84] employed a protocol based on the SICAR (Structure Intracellular Concentration Activity Relationship) index to quantify the intracellular accumulation of a series of fluoroquinolones in individual as well as in a population of *E. coli* cells. Additional molecular simulations rationalized the different accumulation profiles in terms of peculiar interaction patterns established by antibiotics with the DP of this transporter. Specifically, good substrates of AcrB featured multiple and resilient hydrophobic interactions with the DP including the HT, while poor substrates displayed significant hydration that was generally constant in all the DP regions visited by the molecule during the MD trajectory. The authors suggested that, as seen in previous studies, the screening of the interactions with DP residues could not be optimal for triggering allosteric conformational changes needed by AcrB to accomplish its function. More recently, the same authors developed a whole-cell competition efflux assay that allowed them to measure the efficacy of extrusion of clinically used quinolones in populations and individual bacteria. Experiments and computations revealed the efficient competitive action of some quinolones in restoring active concentrations of other fluoroquinolones [85].

### Polyspecificity and multiple entry routes for substrates

As mentioned in the Introduction, the presence of multiple entry pathways in the IMP, each with different sizes and substrate specificities, is another key feature for the recognition and binding of a plethora of compounds with a large spectrum of chemical–physical properties [12, 21]. In AcrB of *E. coli *, up to four channels, named CH1–4, regulate the access of different substrates (Fig. 2). From a computational perspective, only a few studies addressed the link between (poly)specific recognition of substrates and the presence of such multiple entrance gates.

Grimsey *et al.* [86] investigated the binding of the antipsychotic drugs chlorpromazine and amitriptyline to the AcrB transporter of *E. coli* and *Salmonella enterica *. By performing blind docking calculations, they found that the former drug can bind just beneath the CH3 located in the central cavity and proposed to mediate the entry of planar aromatic cations [41]. While both drugs are cationic but non-planar, the molecular core of chlorpromazine was shown to assume a flatter conformation compared to amitriptyline, which could rationalize the preference of the former compound for this entrance.

Ornik-Cha *et al.* explored the binding of several ligands, including polyaromatic compounds, to the AdeB transporter of *A. baumannii* [66]. This pump is also endowed with multiple binding sites and an extraordinarily wide substrate spectrum, which seems even higher than that of AcrB [66, 87–89]. Molecular docking followed by free-energy calculations revealed the binding of ethidium and rhodamine 6G within a crevice nearly equivalent to CH2 found in a new state named L*, having structural features in between the L and T conformations. The results suggested a role for the L* conformational state in initial drug uptake (and in the overall catalytic drug transport mechanism – see below).

## Mechanism of substrate transport

### Studies on IMP transporters

**Fig. 5.**
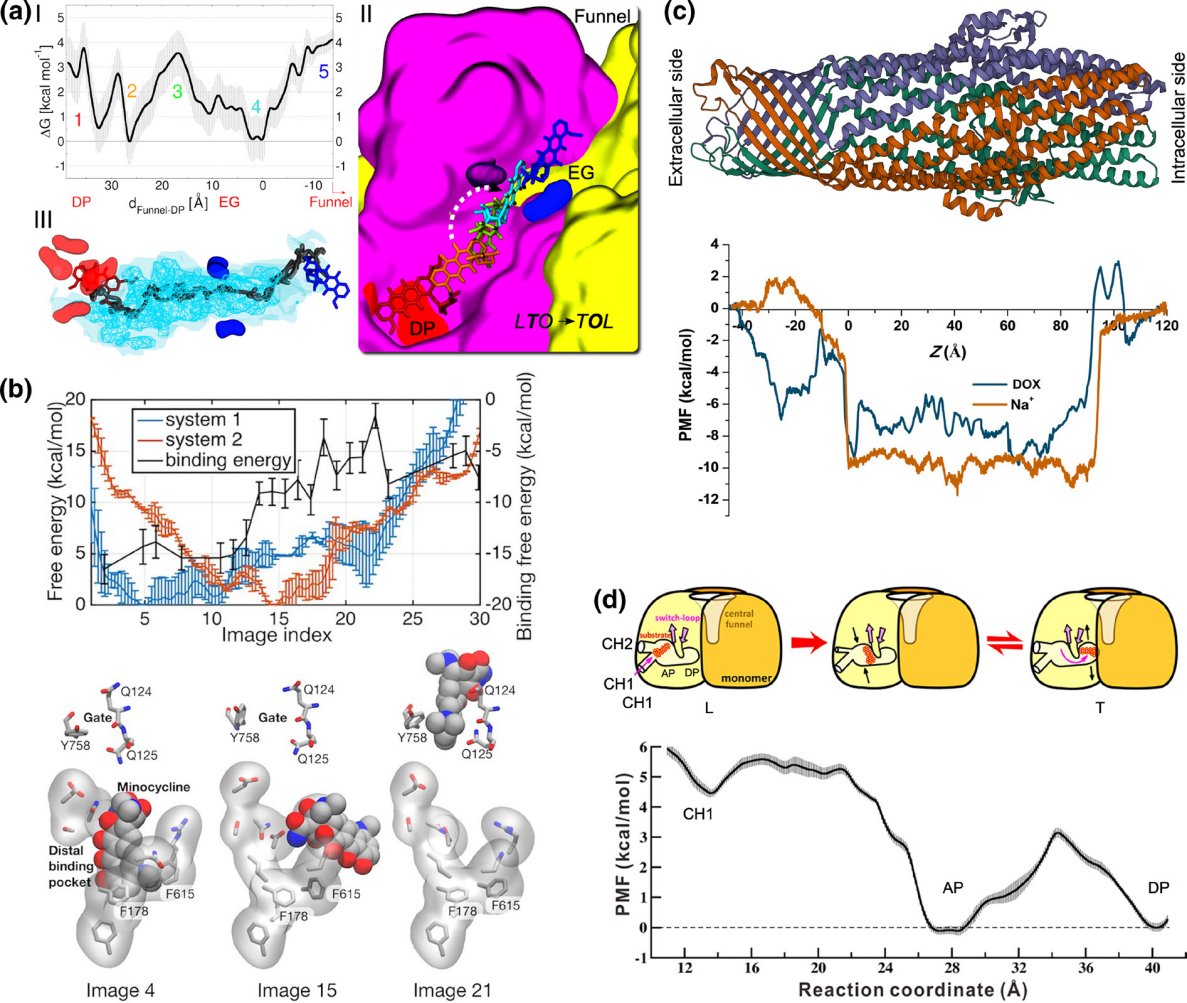
Energetics of substrate transport by RND efflux pumps. (**a**) I. Free energy profile associated with the transport of DOX from the DP to the rear of the EG along the LTO→TOL step of the functional rotation in AcrB. Coloured numbers in the graph identify stages of the transport process, for which the conformation of DOX is shown in II with sticks having the same colour code. II. Representative conformations of DOX along the translocation pathway. III. Water density isosurfaces within the transport channel leading from the DP to the Funnel domain: surfaces corresponding to isovalues of 5 and 1 (with respect to the average value in bulk water) are shown as cyan nets and transparent surfaces, respectively. The positions of DOX at the beginning and at the end of the simulations are shown as red and blue sticks, respectively. The dark grey tube indicates the pathway traced by the centre of mass of the drug during its translocation within the channel. Adapted with permission from [81]. (**b**) Upper panel: dependence of the free energy pathways associated with the translocation of minocycline upon protonation of D408 in AcrB. In system 1 (blue line) D408 is protonated in the O monomer, which is forced to assume the L conformation during biased MD simulations. In system 2 (orange line), D408 is protonated in the T monomer (assuming the O conformation at the end of the simulation). Free energies are referenced to their minima (against the left *y*-axis). The black line indicates the absolute binding free energy of the antibiotic for each image of system 2 (against the right *y*-axis). Lower panel: representative structures of the DP and EG residues (sticks) and of the drug (spheres) are shown for selected images. Adapted with permission from [96]. (**c**) Potential of mean force (PMF) profiles estimated for transporting Na^+^ (orange) and DOX (blue) within TolC, whose trimeric structure (PDB ID: 1EK9 [185]) is shown in cartoons coloured by polypeptide chain. Adapted with permission from [112]. (**d**) Upper panel: cartoon diagram showing the translocation of DOX in AcrB investigated in [91] from CH1 to DP through the AP of AcrB (along with the conformational changes undergone by the transporter during the LTO→TOL step of the functional rotation). Lower graph: PMF profile estimated for the translocation of DOX sketched in the upper panel. Adapted with permission from [91].

While docking and standard MD techniques are often sufficient to investigate the binding of different compounds to the RND efflux pumps, more advanced and customized protocols have been employed to address the conformational changes and the energetics of substrate uptake and transport [12, 42]. The first computational study addressing the functional rotation mechanisms employed biased all-atom MD simulations to mimic the transport of the substrate doxorubicin (hereafter DOX) from the DP of monomer T towards the EG of AcrB (see Fig. 2) [90]. A translocation of ~10 Å towards the EG, induced by a zipper-like squeezing of the DP and a concomitant opening of the EG channel, was observed along the L**T**O→T**O**L transition (protomer bound to the ligand in bold type), although the full extrusion was not covered owing to the short timescale of the simulations (10 ns). The same authors later captured the lubricant action of water molecules that move from the DP towards the EG during the same step of the functional rotation [82]. The role of water molecules in the recognition and transport of substrates is not surprising, considering that the conformational changes occurring in the IMP affect the hydration of its internal channels. In fact, structured water molecules were shown to be crucial to smoothen the interactions between the transporter AcrB and its substrate DOX along the extrusion pathway from the DP to the Funnel domain [81]. As a result, a very low free energy barrier (~3.5 kcal mol^–1^) was associated with the extrusion of the substrate (Fig. 5a). Importantly, water molecules should play a role not only for the binding of unrelated substrates (including inhibitors) to the IMP [34, 35], but also in facilitating substrate diffusion along the extrusion pathway within this protein; that is, polyspecific transport. In other words, hydration seems to be crucial for the fine tuning of protein–substrate interactions behind the Goldilocks effect [78]. Biased all-atom MD simulations were also employed by Zuo *et al.* [91] to mimic the translocation of the same substrate (DOX) from CH1 to the AP and from that site to the DP of AcrB along the **L**TO→**T**OL transition. The authors found that DOX can bind at the CH1 of the T protomer, as well as to the AP and DP with comparable affinities. In addition, they estimated a free energy barrier of ~3 kcal mol^–1^ for the translocation of DOX between these sites, while the displacement from CH1 to the AP appears to be virtually barrierless (Fig. 5d).

Because of the energy transduction function of the proton-relay site (D407/D408), this region remains a focus of substrate-transport studies. Yamane *et al.* demonstrated for the first time by computational means the allosteric link between protonation/deprotonation of residues in the TM region of AcrB and conformational rearrangements occurring at the periplasmic region of the protein [92]. By simulating all possible combinations of protonation states of D407 and D408 in the O monomer, the authors suggested that the combination D407 deprotonated/D408 protonated is compatible with the structure of the O state. Indeed, deprotonation of the latter aspartate is accompanied by a large rearrangement of the TM region (although, due to the relatively short simulation time, the actual structural movements accompanying the entire functional cycle were not observed). Based on their findings, the authors proposed the ‘one-proton’ model, in which only D408 drives the conformational cycling of AcrB.

Eicher *et al.* [25] suggested a different protonation state, whereby both D407 and D408 are protonated, as the most likely for the O state. This conclusion was based on a combination of experiments and simulations performed to investigate the transport mechanism in the wild-type and in several PMF-deployed (inactive) variants of AcrB. It was shown that the functional rotation mechanism is mediated by two remote alternating-access conformational cycles within each monomer, one occurring in the periplasmic domain and regulating the transport of substrates, and another one occurring in the TM region and regulating the flux of protons across conformation-dependent water channels. Access to the proton relay site occurs from the periplasm in the T state, as opposed to the L and O states, where a water wire extends to the cytoplasm. This finding agrees with an earlier publication by Fischer and Kandt [93], who identified continuously hydrated regions extending from the TM to the cytoplasm and up to the periplasm in the L and T monomers of AcrB. Based on these findings, the authors also postulated proton uptake to occur in L and/or T or in an intermediate conformation in between T and O, and a proton release event accompanying the O to L transition.

Yue *et al.* [94] explored the protonation states and conformational dynamics of the transmembrane domain of AcrB using membrane hybrid-solvent continuous constant pH MD simulations (CpHMD) [95]. The authors found that both D407 and D408 are deprotonated in L and T states, while D408 is protonated in the O state. In particular, the p*K*_a_ of D408 increases by two units along the T to O conformational change, which suggests that this amino acid takes up one proton and thus supports the study by Yamane *et al.* [92]. Furthermore, the authors reported that proton release from D408 in the O state caused significant conformational changes (namely, lateral and vertical movements of TM helices), which not only facilitates salt-bridge formation between D408 and K940 but also rearranges the side chains of additional essential residues. Matsunaga *et al.* [96] performed free-energy calculations to study the coupling between the functional rotation and the proton translocation and suggested that transient deprotonation/protonation of D408 in the transmembrane region of AcrB can drive functional rotation and the extrusion of a drug. They found that the extrusion of minocycline from the DP to the EG is associated with a non-negligible free-energy barrier (Fig. 5b), suggesting that: (i) the release of the drug is the bottleneck step of the functional cycle, and (ii) more than one drug per monomer could be needed to progress along the whole functional rotation.

Jewel *et al.* [97] also conducted MD simulations using the hybrid coarse-grained (CG) PACE [98] force field (in which a united-atom-based protein model is coupled with the MARTINI [99] water/lipid environment) to investigate how the protonation state of D407/D408 in AcrB influences the conformational dynamics of the porter domain of the protein. They found that the asymmetric (functional) structure of AcrB is stabilized by protonation of D408 in the O monomer, which evolves toward the L state upon deprotonation. Furthermore, simulations supported the symmetric LLL conformation in the absence of substrates.

In a subsequent study, the same authors employed a similar computational protocol to address how these conformational changes in AcrB were affected also by the binding of indole to the porter domain of the T monomer [100]. They reported proton-dependent conformational changes whereby the presence of indole and protonation at D407 or D408 facilitate conformational changes from the T to the O state, while both the L and O monomers assume a conformation close to L at the end of the simulation. Furthermore, biased MD simulations were performed to mimic indole transport in protonated systems.

The transport of substrates (namely minocycline) along the LTO→TOL transition was also studied by employing a CG model of the pore domain of AcrB and of the antibiotic [101]. Key findings of this investigation were that the allosteric coupling stabilized the asymmetric structure of the protein with one antibiotic molecule bound and that the dissociation of this molecule induced a conformational change towards the symmetric structure of AcrB representing its resting state in the absence of substrates [102]. Furthermore, the authors performed a MD study that led to the proposal of the protonation of the drug-bound protomer as the driving force promoting the functional rotation of AcrB and the simultaneous export of the drug. In a different study, Feng *et al.* analysed the interaction of AcrB with three substrates (rifamycin, erythromycin and minocycline) and reported a unidirectional peristaltic movement of all compounds [103].

In particular, rifamycin and erythromycin, initially bound to the AP of the L monomer, were found to move towards the DP following a shift in the switch-loop, while minocycline, initially bound to the DP of the T monomer, moved towards the EG. However, the movement of compounds was relatively shorter possibly due to the relatively short simulation time (20 ns). The authors further investigated the impact of mutations G616P and G619P on the switch-loop, showing that they prevent its movement, and confirming the key role of its flexibility in the transport of compounds bound to the AP [37].

**Fig. 6.**
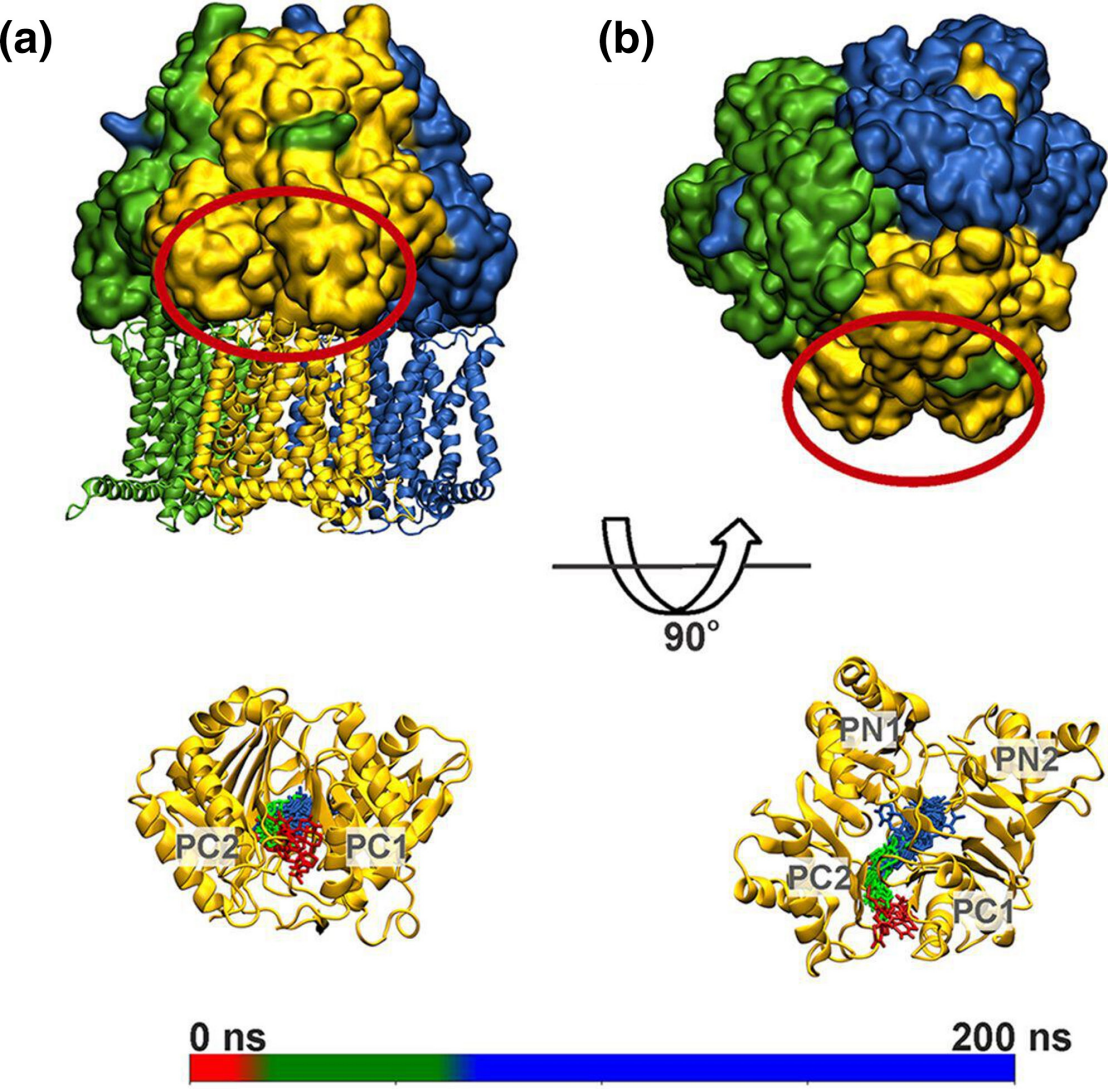
Uptake of a progesterone molecule by the T monomer of MtrD (A, B: side and top views, respectively). The L, T and O protomers of the transporter are coloured green, gold and blue, respectively, and the periplasmic region of protomer T is circled in red. The lower panels show the position of progesterone within the T protomer of MtrD along a 200 ns long MD simulation (snapshots taken every 10 ns are shown in sticks coloured according to the simulation time as rendered in the scale bar. Taken with permission from [83].

Recently, the RND transporter MtrD of *N. gonorrhoeae* was investigated by several authors. Brown and co-workers investigated the binding of the substrate progesterone to this transporter by means of molecular docking and MD simulations. The latter showed how this compound moves from a region approximately corresponding to CH2 to the AP, and from there further towards the DP (Fig. 6) [83]. O’Mara and co-workers also employed MD simulations and mutagenesis experiments [104] to provide molecular insights into the allosteric coupling between substrate binding sites and protonation states controlling the transition from symmetric (resting) to asymmetric (active) MtrD conformations. Their study, which represented the first computational description of the transport of substrates by this protein, supports a functional rotation mechanism also for MtrD.

In the same year, Ammerman *et al.* [105] studied substrate (azithromycin) and non-substrate (streptomycin) interactions and dynamics with the same RND transporter using free and targeted MD simulations. The authors determined the spontaneous diffusion of azithromycin through CH2 and found that it takes an unexpected pathway along PN1, bypassing the DP altogether. By contrast, streptomycin transport is not seen in similar simulations, supporting the finding that this compound is not a substrate nor is it likely to be a potent MtrD inhibitor. The authors’ findings also support the slow substrate diffusion in the absence of a proton-relay network and the slow migration from AP or DP, as seen in other computational investigations reported above.

Substrate uptake pathways were earlier investigated by Yao *et al.* [106] by means of CG MD simulations of AcrB. The authors identified three putative pathways, one starting from CH2 and two from CH1. Site-directed mutagenesis experiments validated the pathway starting at CH1, thus supporting its relevance *in vivo*, as also confirmed by subsequent publications [21]. Importantly, this study proposed that the preferred uptake pathway depends upon mass, hydrophobicity and hydrophilicity of compounds: drugs that are bulky and/or feature large hydrophilic surfaces enter mostly through CH2, whereas small and/or hydrophobic compounds are preferentially transported through CH1. Tam *et al.* [39] employed extensive molecular docking calculations to demonstrate that carboxylated drugs, such as fusidic acid and hydrophobic β-lactams, can enter AcrB via CH1/CH4 channels. Based on the computational data, the authors proposed that membrane-embedded drugs, after binding to the entry gates, are first oriented by the AcrB PN2 subdomain and finally transported via a PN2/PC1 interface pathway directly toward the DP. Functional and structural characterization of several AcrB variants corroborated *in silico* data, and the recent availability of co-crystal structures of fusidic acid with additional AcrB intermediates confirmed the possibility of such a pathway as a true transport route [30].

Intensive *in silico* mutagenesis studies have been performed to understand molecular details of substrate recognition and transport mechanisms in RND efflux pumps. In one study [77], the effects of the F610A substitution in AcrB, shown to impair the activity of several antibiotics due to delayed efflux [107, 108], were assessed by means of docking calculations coupled with standard and biased all-atom MD simulations. The authors suggested that the removal of bulky phenylalanine within the HT favoured the sliding of substrates (namely DOX) within this cage, leading to better packing of the drug inside that region. This resulted in a higher affinity of DOX for the protein and in an increased dwelling time within the HT, which would hamper the displacement of the compound towards the EG during the L**T**O→T**O**L transition and eventually inhibit AcrB functional dynamics. Similar findings were later reported for minocycline [16]. These outcomes are reminiscent of the ‘Goldilocks effect’ recently proposed to explain substrate selectivity of *P. aeruginosa* MexY and MexB transporters [78]. Another key mutation within the DP of AcrB is G288D, which was reported to decrease ciprofloxacin susceptibility by increased efflux, but increased the susceptibility of other drugs (like DOX) via decreased efflux in a clinical strain of *Salmonella Typhimurium* [109]. MD simulations suggest that this mutation affects the structure, dynamics and hydration properties of DP of AcrB and thereby influences in different ways the binding propensities of different compounds. Besides the two main binding pockets of the RND transporters, the switch-loop has been shown to be crucial for substrate binding and transport [65, 110]. With the help of MD simulations, Müller *et al.* [37] investigated the impact of point mutations in the PC1-proximal and PN1-proximal sides of this loop in AcrB (previously investigated experimentally also by Yamaguchi and co-workers [40]). They found that the G614P and G616P variants reduced the flexibility of the loop on the PN1-proximal side, while the G619P and G621P variants enhanced the flexibility of the PC1-proximal side. This work confirmed that the switch-loop contributes to the promiscuous adaptation of drugs in the AP and DP, broadening the substrate scope.

Nikaido and co-workers performed active efflux measurements and computer simulations to study the synergistic effect of different molecules, including apolar solvents such as benzene and cyclohexane, on the transport of cephalosporins by AcrB [111]. Molecular docking and MD simulations of benzene and nitrocefin in complex with AcrB demonstrated that these compounds can bind simultaneously to the DP. Moreover, compared to its position in the binary complex with AcrB [36], the antibiotic is displaced towards the EG in the presence of benzene, which could facilitate its extrusion through enhanced functional cycling of the transporter.

### Transport through the OMPs

Few computational studies also focused on substrate transport across the OMP. Wang and co-workers estimated the free-energy profile associated with the translocation of DOX and Na^+^ within the *E. coli* TolC channel [112]. They found a roughly flat profile along the middle region of TolC, while permeation barriers associated with substrate-dependent gating mechanisms were found at both the periplasmic and the extracellular ends of the protein (Fig. 5c). The interplay between conformational changes in TolC and the interaction between substrate and protein determines these free-energy profiles. The same authors performed MD simulations of the wild-type and of six variants of TolC to explore its conformational dynamics [113]. They reported that all mutations except Y362F favoured the opening of the TolC periplasmic gate. The intermediate states were associated with asymmetric conformations of this region of the channel, while the most and the least open states were more symmetric. Finally, the authors showed that the closed state of the periplasmic gate becomes preferred at lower pH values. Schulz and Kleinekathöfer [114] performed MD simulations to investigate the transition between the closed and open conformations of TolC. A partially open conformation was generated from a closed one by means of a double point mutation that was experimentally shown to weaken salt bridges and H-bonds at the constricting ring.

## Mechanisms of inhibition

### Inhibition of the IMP by small molecule binding

**Fig. 7.**
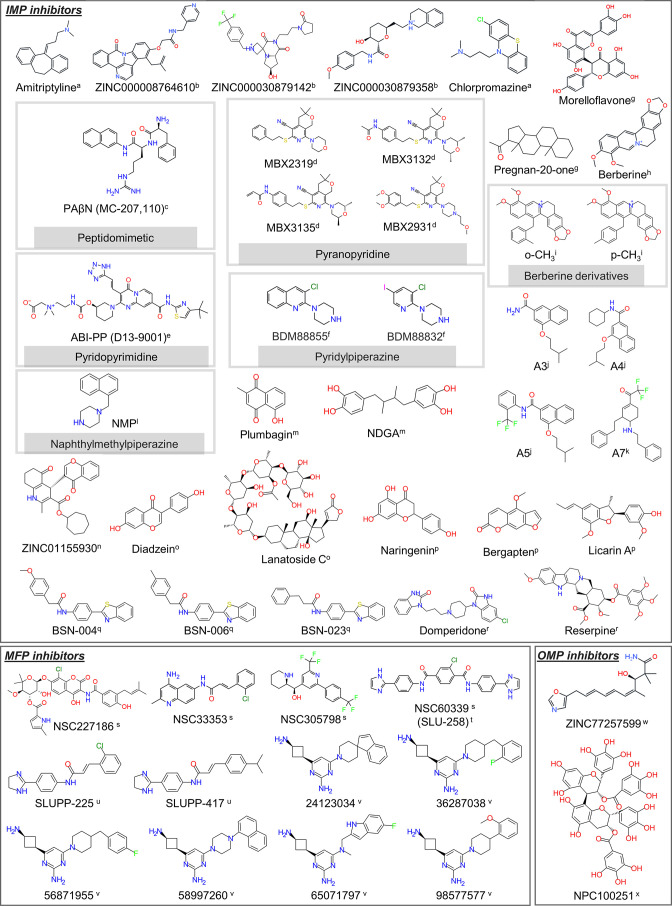
Chemical structures of selected EPIs inhibiting RND pumps in Gram-negative bacteria. Taken with permission from [83]: a [86], b [146], c [123], d [34, 129], e [33], f [148], g [137], h [138], i [140], j [136], k [142], l [156], m [119], n [156], o [118], p [141], q [143], r [144], s [150], t [152], u [153], v [155], w [156], x [157].

Various molecules, including medicinal plant extracts and synthesized compounds, have been designed to bind RND efflux pumps at a particular site and interfere with the active efflux of antibacterial agents [46, 115–121]. Some of these efflux pump inhibitors (EPIs) are reported in Fig. 7.

Most EPIs bind to the periplasmic domain of one or more RND transporters (Table 1). The first discovered EPI, phenylalanine-arginine β-naphthylamide (PAβN [122]), is supposed to act either by competitive binding to the RND transporter [36, 123] or by interfering with the functional dynamics of the transporter [124] (although a recent report provided evidence of a target site within the LPS layer of *E. coli* and proved that destabilization of the membrane by this EPI contributes to its drug-sensitizing potency [125]). Takatsuka *et al.* [32] performed molecular docking to investigate the binding of EPIs PAβN and 1-(1-naphthylmethyl)-piperazine (NMP) to the DP of AcrB. They found that NMP binds to the cave region of this site, while PAβN binds in a mixed way, contacting both that region and the upper groove of the DP. A subsequent study characterized the binding of these EPIs by means of MD simulations and estimated the free energies of binding to the DP AcrB [36]. Both PAβN and NMP moved away from their docking poses during the MD simulations, straddling the switch-loop and thus altering its conformational flexibility that is crucial for the correct functioning of the pump [16, 33, 35, 40]. Namely, the authors suggested that, by reducing the flexibility of the switch-loop, this interaction can interfere with the efflux of substrates. These results agree with MD simulations of switch-loop AcrB mutants shown to hinder drug export through altered dynamics of the same loop [37, 103]. A similar finding was also reported by Passarini *et al.* [126], who investigated by means of docking calculations and short MD simulations the mode of action of small cationic peptides (designed based on similarity to known active peptides and EPIs such as PAβN) improving the activity of novobiocin against a susceptible *E. coli* strain. The authors found that the most potent peptides can bind either at the DP or at the AP/DP interface of AcrB, interacting with the switch-loop in a similar way as reported for PAβN [36]. Nikaido and co-workers determined quantitatively the efflux kinetics of PAβN and of its homologues alanine, arginine and phenylalanine β-naphthylamides [127]. Computer simulations supported the hypothesis that inhibition of AcrB by PAβN is due to its binding at a region including the HT; in this way, the EPI interferes with the binding of substrates to the upper groove of the DP. The same hypothesis was put forward for Phe-β-naphthylamide, which competes with nitrocefin for binding to the same region of the DP (namely, the groove). In contrast, stimulators of efflux such as Ala- and Arg-β-naphthylamides were shown to bind simultaneously with nitrocefin to the DP, acting as efflux enhancers in a way similar to that reported earlier for benzene [111]. Based on their findings [111, 127], the authors proposed that the loose binding of substrates explains the positive cooperativity and sigmoidal kinetics reported in previous studies [1, 2]. A more recent study combining simulations and hydrogen/deuterium exchange experiments suggested that PAβN could also inhibit AcrB by restricting DP dynamics [124]. Simulation data demonstrated that a typical substrate (ciprofloxacin) and the EPI can bind simultaneously to different DP subpockets and that the latter compound inhibits the protein by altering the functional dynamics of the substrate translocation pathway.

**Table 1.** Available inhibitor and substrate-bound experimental structures

Class of inhibitor	Representative compound(s)	Region of binding	Intrinsic antibiotic activity (MIC)*	Efflux substrate	PDB ID	Mechanism(s) of action (hypothesis)
Peptidomimetic	PAβN (MC-207110 dihydrochloride) [122]	CC/AP within the RND transporter (LTO/L states) [32, 36, 123]	512 µg ml^−1^ [122]	Piperacillin, cefotaxime, ceftazidime, FQs, macrolides, TETs [186]	1T9Y [123]	Competitive inhibition [127, 129]/altered functional movements [124]/membrane permeabilization or other target [125, 186]
Pyridopyrimidine	ABI-PP (D13-9001) [187]	HT within the RND transporter (T state) [33]	–	FQs, β-lactams	3W9H (bound to AcrB), 3W9J (bound to MexB) [33]	Competitive inhibition [33]
Pyranopyridine	MBX3132 [131]	HT within the RND transporter (T state) [34]	>100 µg ml^−1^ [131]	FQs, PIP	5ENO (MBX2319), 5ENP (MBX2931), 5ENQ (MBX3132), 5ENR (MBX3135) [34]	Competitive inhibition [34]
Pyridylpiperazine-based	BDM88832 [148]	Transmembrane domain of RND transporter (L state) [148]	>500 µM >250 µM	Pyridomycin	7OUK (BDM8885), 7OUL (BDM88832) [148]	Allosteric inhibition [148]
Antidepressant	Amitriptyline [86]	HT within the RND transporter (T state) [86]	–	CIP, NAL, norfloxacin [86]	–	Competitive inhibition/altered functional movements [86]
Antipsychotic	Chlorpromazine [86]	HT within the RND transporter (T state) [86]	256 g ml^−1^	CIP, NAL, norfloxacin [86]	–	Competitive inhibition/altered functional movements [86]
Pyridoindole-based	ZINC000008764610 [146]	DP within the RND transporter (T state) [146]	–		–	
Piperazine-based	ZINC000030879142 [146]	DP within the RND transporter (T state) [146]	–		–	
	1-(1-Naphthylmethyl)piperazine (NMP) [188]	DP within the RND transporter (T state)	–	CHL, linezolid [188]	–	Non-competitive/competitive
Hydroisoquinoline	ZINC000030879358 [146]	DP within the RND transporter (T state) [146]	–		–	
Phytochemicals	Morelloflavone [137]	CH2/TM and AP within the RND transporter (T state) [137]	–	CIP [137]	–	Possible antibiotic competitors for MexB (four-fold decrease in MIC of CIP) [137]
	Pregnan-20-one [137]	CH2/TM within the RND transporter (T state) [137]	–		–	Possible competitive inhibition (four-fold decrease in MIC of CIP) [137]
	Berberine [138]	DP within the RND transporter (T state) [138]	–	Tobramycin [138]	–	Competitive inhibition [138]
	Naringenin [141]	HT within the RND transporter (T state) [141]	–		–	
	Bergapten [141]	HT within the RND transporter (T state) [141]	–		–	
	Licarin A [141]	HT within the RND transporter (T state) [141]	–		–	
	Plumbagin [119]	DP within the RND transporter (T state) [119]	128 µg ml^−1^	ERY, CHL, TPP [119]	–	Probably competitive inhibition (decrease in MIC of ERY) [119]
	Lanatoside C [118]	AP within the RND transporter (L state) [118]	–	CAR and LEV [118]	–	
	Diadzein [118]	CC within the RND transporter [118]	–	CAR and LEV [118]	–	
	Reserpine [144]	DP within the RND transporter (T state) [144]	NI	LEV, CIP [144]	–	Probably by competitive binding (reduce the MIC values of both LEV, CIP in *E. coli *) [118]
Antiviral agent	Nordihydroguaretic acid (NDGA) [119]	DP within the RND transporter (T state) [119]	512 µg ml^−1^	ERY, CHL, NOV, TET, TPP, NAL [119]	–	Probably by competitive binding (MIC, Nile red efflux assays) [119]
4-Substituted 2-naphthamide derivatives	A3† [136]	DP within the RND transporter (T state) [136]	–	ERY, CHL, TPP [136]	–	Dissipation of the proton motive force [136]
Hexahydroquinoline-based	ZINC01155930 [132]	OMP protein (AdeABC) [132]	--	–	–	
2-Substituted benzothiazoles	BSN-004 [143]	HT within the RND transporter (T state) [143]	256 µg ml^−1^	CIP [143]	–	Competitive Inhibition [143]
Hydroxybenzimidazole-based	Domperidone [144]	DP within the RND transporter (T state) [144]	NI	LEV, CIP [144]	–	Probably by competitive binding (reduce the MIC values of both LEV, CIP in *E. coli *) [144]
Aminocoumarin	NSC227186 (chlorobiocin) [150]	Hinge and membrane-proximal site of MFP [150]	>50 µM	NOV, ERY [150]	–	Altered functional interactions between AcrA and AcrB [150]
Aminoquinoline	NSC33353 [150]	Hinge and membrane-proximal site of MFP [150]	200 µM	NOV [150]	–	Altered functional interactions between AcrA and AcrB [150]
Piperidinyl-based/antimalarial agent	NSC305798 [150]	Hinge and membrane-proximal site of MFP [150]	50 µM	NOV [150]	–	Altered functional interactions between AcrA and AcrB [150]
Phthalanilide derivatives	NSC60339 (SLU-258) [150]	Hinge and membrane-proximal site of MFP [150]	>200 µM	NOV [150]	–	Disruption of pump assembly/structural changes in AcrA
Dihydroimidazoline derivatives	SLUPP-225 [153]	MFP [153]	25 µM	NOV, ERY [153]	–	Disruption of efflux pump assembly [153]
2-Aminopyrimidine	24123034 [155]	MP and β-barrel domain (Site II/III†) of MFP/HT within the RND transporter (T state)	≥200 µM	NOV, ERY	–	Related to binding to HT of RND transporter (detected by SPR binding assays)/promote efflux of H33342 (RND substrate)
Oxazole	ZINC77257599 [156]	OMP (AdeABC of *A. baumannii *) [156]	–	–	–	
Benzenediol derivative	NPC100251 [157]	OMP (various pumps) [157]	–		–	Proposed to compete with substrate antibiotics to bind with OMPs [157]

*The reader is referred to the corresponding publication to get information about the specific bacterial strain(s) used to obtain MIC values.

†Please refer to Fig. 9.

NI, no inhibition (below measurable limit); MP, membrane proximal; MFP, membrane fusion proteins; TPP, tetraphenylphosphonium; ERY, erythromycin; NOV, novomycin; CHL, chloramphenicol; TET, tetracycline; FQ, fluoroquinolones; CIP, ciprofloxacin; LIV, levofloxacin; CAR, carbenicillin; NAL, nalidixic acid.

Zuo *et al.* [128] performed computer simulations to provide insights into the functioning of the EPI D13-9001, which was shown to bind the HT in AcrB and MexB [33]. The authors performed biased MD simulations to compare the free energy required to displace this EPI and DOX from the DP along the L**T**O→T**O**L step of the functional rotation cycle. They found that the larger affinity of the EPI towards the HT reflects delayed dissociation of this compound from the DP, as compared to DOX.

In another work, Vargiu *et al.* compared the binding modes of PAβN, NMP, the pyridopyrimidine D13-9001 (active against AcrB and MexB transporters) and the pyranopyridine MBX2319 (potent against RND pumps of *Enterobacteriaceae* species) to the DP of AcrB using docking, MD simulations and free-energy calculations [129]. According to *in silico* data, MBX2319 and D13-9001 have higher affinities to the DP than the substrate minocycline; the authors suggested that all inhibitors hinder binding of the substrate to the upper part of this pocket, either by reducing the space available or by blocking access to this region. Rahman and co-workers [130] combined homology modelling, molecular docking, MD simulations and free-energy calculations to predict the binding mode of the broad-spectrum peptidomimetic inhibitor PAβN in AdeB from *A. baumannii *. They found that this EPI does not interact specifically with the AP (differently from what was previously found in experimental structures of AcrB in a symmetric LLL conformation [123]), while it binds strongly to the DP, consistently with the previous prediction made for the same inhibitor on AcrB [32, 36]. The authors suggested that this inhibitor exploits the hydrophobic microenvironment within the HT of the DP to lock the monomer in the T conformation, inhibiting the peristaltic mechanism.

Sjuts *et al.* [34] combined biochemical and structural experiments with computer simulations to investigate the molecular basis for pyranopyridine-based inhibition of AcrB. In their study, three derivatives of MBX2319 were investigated (Table 1), all more potent than the original inhibitor [131]. All the compounds were found to bind to the HT (supporting the hypothesis disclosed in [129]), forming extensive hydrophobic interactions and interfering with the binding of substrates. Importantly, an intricate protein- and water-mediated hydrogen bond network was found to correlate with the improved potency of the MBX derivatives.

Several studies identified EPIs by screening databases of natural and synthetic compounds. Verma *et al.* [132] performed high-throughput virtual screening of about 160 000 medicinal compounds to identify putative EPIs of AdeB. Of these, about 100 compounds were selected and their affinity to the transporter was estimated by means of Molecular Mechanics-Generalized Born Surface Area (MM-GBSA) calculations [133], exploiting the generalized Born implicit solvation theory to estimate free energies of binding.^1^ Finally, the interaction of the lead compound with AdeB was further validated by MD simulations. This protocol led to the identification of ((4*R*)−3-(cycloheptoxycarbonyl)−4-(4-etochromen-3-yl)−2-methyl-4,6,7,8-tetrahydroquinolin-5-olate) (ZINC01155930, Fig. 7) as a putative inhibitor for AdeB.

Recently, machine learning algorithms were applied by Mehla *et al.* to identify properties of small peptidomimetic molecules that correlate with efflux avoidance and inhibition in *P. aeruginosa* [134]. Intrinsic properties and descriptors of the interaction with MexB were identified as relevant for EPI activity, and for discriminating between efflux avoiders and inhibitors. The power of these predictors was demonstrated against a library of traditional antibiotics and compound series and by generating new inhibitors of MexB. In another study, Aparna *et al.* [118] exploited the fact that substrates of the efflux pumps AcrAB-TolC and MexAB-OprM share a common pharmacophore feature map to single out non-substrate EPIs of AcrB and MexB. The authors performed high-throughput virtual screening of a database of phytochemicals against AcrB and MexB and selected hits by excluding compounds that matched with any of the common pharmacophore models generated using known efflux substrates. The inhibition potency of putative leads was validated by a checkerboard synergy assay and ethidium bromide accumulation assay. Lanatoside C and daidzein were finally selected as promising EPIs effective for use in combination therapy against MDR strains of *E. coli* and *P. aeruginosa *.

Several studies searched for EPIs by virtually screening ‘random’ libraries of compounds. Wang *et al.* [135] employed molecular docking to show that the compound A3 ([4-(isopentyloxy)−2-naphthamide]) (Fig. 7), an analogue of 2-napthamide, binds to the DP of AcrB and forms critical contacts with key residues of the HT, in a very similar way to MBX2319. Furthermore, using *in silico* Structure Activty Relationships (SAR), they substituted the fourth position of 2-naphthamide and developed two compounds (A4 and A5) with improved activity in synergy with erythromycin and chloramphenicol [136].

In another study, two novel EPIs, morelloflavone and pregnan-20-one (Fig. 7), were identified by Mangiaterra *et al.* [137] by virtually screening databases of natural compounds and comparing their common pharmacophoric fingerprints. Importantly, *in vitro* experiments confirmed the ability of both compounds to reduce the MIC and increase the bacterial killing by ciprofloxacin. The same authors later explored the binding of ligands from the database of natural compounds ZINC to the MexY transporter, responsible for aminoglycoside resistance in *P. aeruginosa* [138]. Docking calculations revealed that the alkaloid-based natural inhibitor berberine can bind with high affinity to MexY as compared to the aminoglycoside substrate tobramycin. Based on their results, the authors proposed that berberine acts as a competitor of the antibiotic and there by prevents its extrusion. The hypothesis was confirmed in a subsequent study on both planktonic and biofilm cultures of *P. aeruginosa *, confirming the involvement of MexXY-OprM in the tolerance of this bacterium to tobramycin [139]. In particular, the authors performed molecular docking calculations of berberine on different variants of MexY showing either reduced tolerance or reduced MIC, or both, or wild-type phenotype. They observed a reduction of berberine estimated binding affinity on the latter two MexY variants, due to the lack of H-bond interactions present in the two former strains. Based on these findings, the authors suggested that a hydrophilic contribution is required for a strong berberine binding, explaining the lack of inhibitory activity observed in strains where only a non-specific binding due to hydrophobic interactions can be established.

Finally, in the search for berberine derivatives with enhanced potency, the same lab estimated the binding affinity of three aromatic substituents to the three polymorphic sequences of MexY found in *P. aeruginosa* (PAO1, PA7 and PA14) [140]. All derivatives were able to bind to MexY, although with different modes depending on the substituents and on the specific transporter polymorphism. This mechanism of action was confirmed by *in vitro* assays, showing a strong MIC reduction and a greater killing effect after exposure to the combinations of 13-(2-methylbenzyl)- and 13-(4-methylbenzyl)-berberine with tobramycin against the tobramycin-resistant strain PA7, a milder synergy against PAO1 and PA14, and no synergy against the Δ*mexXY* strain K1525.

Ohene-Agyei *et al.* [119] identified plumbagin and nordihydroguaiaretic acid as promising EPIs of AcrAB-TolC by performing virtual screening and bioassays on a database of phytochemicals. Oyedaara *et al.* [141] screened 71 phytochemicals found in medicinal plants for their potential as EPIs of *S. enterica* AcrB using molecular docking and MD simulations. Naringenin, 5-methoxypsoralen and licarin A were identified as putative EPIs based on their strong binding to the DP and HT regions of AcrB, whose stability was confirmed by MD simulations. Based on simulation data, licarin A demonstrated the highest inhibitory potential. Silva *et al.* investigated the potential of a series of tetrahydropyridine derivatives as inhibitors of AcrB using a novel computational protocol complemented by *in vitro* experiments [142]. They discovered a few compounds (a representative one, A7, is shown in Fig. 7) that are substrates and EPIs of the transporter and proposed competitive binding as their putative mechanism of action. Yilmaz *et al.* [143] reported another successful *in silico* discovery of EPIs by screening 2-substituted benzothiazoles as potential EPIs able to restore the antibacterial activity of ciprofloxacin. Among the various compounds, a few (BSN-004, BSN-006 and BSN-023) topping the list with clinically significant EPI activity were found to bind to the DP of AcrB, possibly interfering with the recognition of substrates. In support of this hypothesis, compounds BSN-006 and BSN-023 featured binding free energies (estimated to be approximately −18.0 and −12.7 kcal mol^–1^, respectively) higher than that of ciprofloxacin (−10.2 kcal mol^–1^) towards this site. This confirms competitive inhibition as a putative mechanism of action, in contrast to BSN-004, which might act as an uncompetitive inhibitor by steric hindrance.

An *in silico* drug repurposing study reported that domperidone reversed resistance to levofloxacin and ciprofloxacin in *E. coli* due to a strong interaction with the HT of AcrB [144]. The reversal of resistance potency in an MDR *E. coli* strain and the binding affinity towards AcrB were both greater than those of the known AcrB inhibitor reserpine, suggesting that domperidone can be used in conjunction with antibiotics to treat infections caused by MDR *E. coli* strains [145]. In a similar spirit, Grimsey *et al.* [86] employed blind docking calculations and MD simulations to show that chlorpromazine and amitriptyline (Fig. 7) are substrates and inhibitors of AcrB in *E. coli* and *S. enterica *. A high number of high-affinity poses were detected within the DP of monomer T, in close interaction with the HT. Subsequent docking calculations of typical AcrB substrates performed on the most stable AcrB-chlorpromazine and AcrB-amitriptyline complexes showed that these EPIs impede AcrB-mediated efflux by interfering with substrate binding.

A recent study on the RND pump MtrCDE of *N. gonorrhoeae* used a comprehensive pharmacophore-based approach, induced fit docking, MD simulations and MM-GBSA calculations to identify EPIs with improved pharmacology/safety profiles [146]. As a result of their investigation, five non-toxic bioactive chemicals (Fig. 7) extracted from the ZINC database [147] were identified as hits with binding affinity towards MtrD higher than that of the known inhibitors PAβN, D13-9001 and MBX2319. In view of their good physiochemical/pharmacokinetic profiles, these compounds could be used for the discovery of novel EPIs against *N. gonorrhoeae *.

**Fig. 8.**
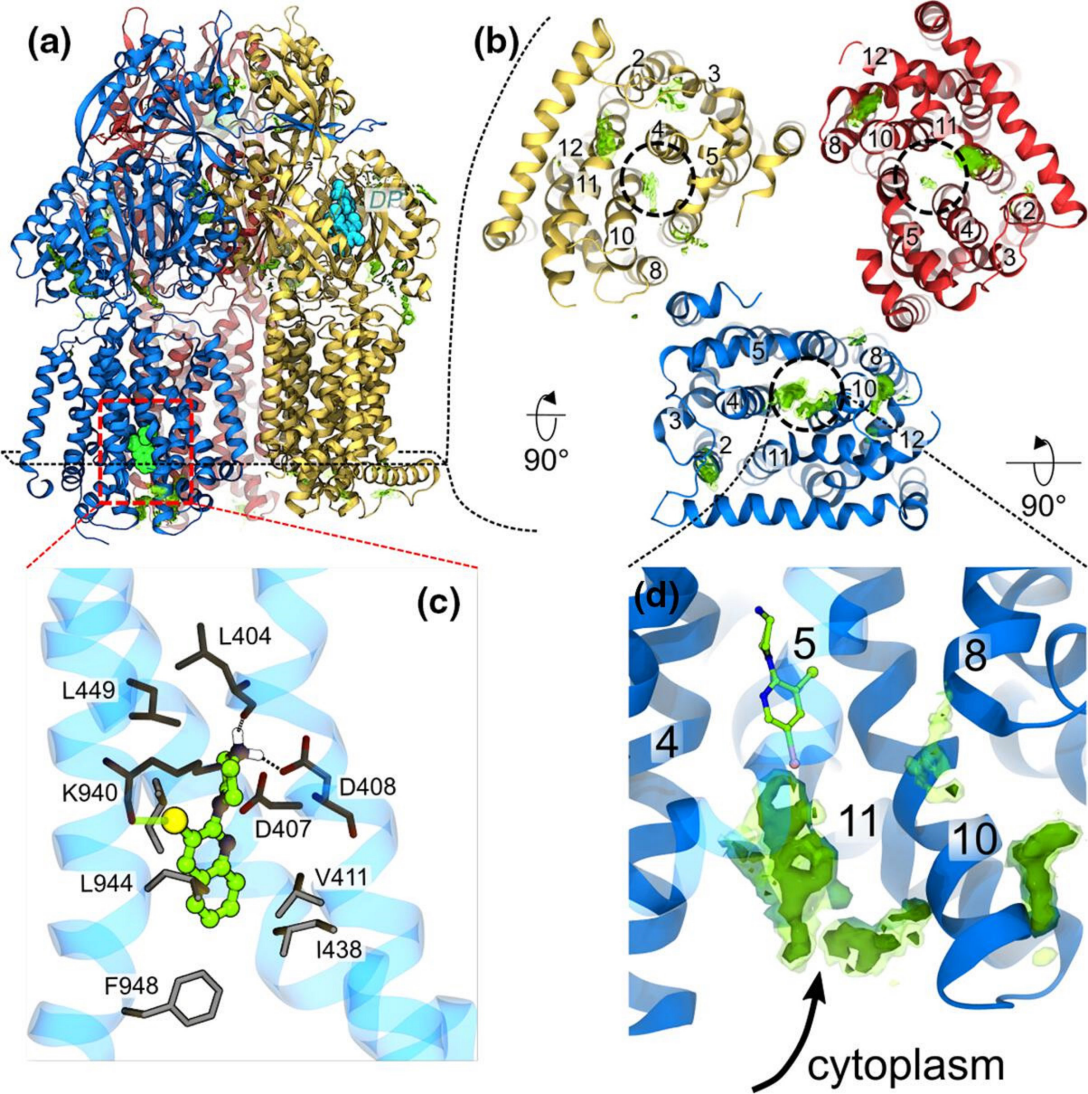
Binding of the compounds BDM88832 and BDM88855 to AcrB. (**a**) Side view of the binding hotspots (green surfaces) of BDM88832 as seen in all-atom MD simulations of 100 compounds placed in a water solution box enclosing AcrB embedded in a phospholipid bilayer. The L, T and O protomers are shown as blue, yellow and red cartoons, respectively, and the X-ray crystallographic conformations of BDM binding to the TM region and of MBX3132 binding to the DP are shown as spheres coloured cyan and green, respectively. (**b**) BDM88832 density distribution viewed from the cytoplasmic side (only TM domains are shown for clarity). Dashed circles on each monomer identify the location of the experimental binding site, and relevant TM helices are labelled. (**c**) Enlarged view of the inhibitor binding site showing interacting residues (sticks coloured by atom type: C, N, O in grey, blue and red, respectively) within 3 Å from BDM88855 (CPK coloured by atom type: C, N, I and Cl in green, blue, pink and yellow, respectively). The H-bonds between the piperazine ring and the carbonyl atom of L404 and the side chain of D408 are shown with dashed lines, while the halogen bond between the inhibitor chlorine atom and the carbonyl oxygen of K940 is shown by a transparent green line. (**d**) Enlarged side view of the L protomer showing accumulation of BDM88832 from the cytoplasm during MD simulations. The X-ray conformation of BDM88832 is shown for reference in CPK representation coloured by atom type (C, N, I and Cl in green, blue, pink and yellow, respectively). The TM helix 5 is shown transparently for clarity. Data extracted from five independent MD simulations of 2 μs each; transparent and solid green surfaces represent iso-values of 3 and 5, respectively. Adapted with permission from [148].

Recently, allosteric inhibition of the RND transporter AcrB was achieved through EPIs containing a pyridylpiperazine scaffold that bind to a new site centred around catalytic residues (D408, D407, K940 and R971) of the proton relay pathway within the transmembrane domain of the L protomer (Fig. 8) [148]. X-ray crystal structures showed that the protonated piperazine moiety of the pyridylpiperazine-based EPIs makes critical salt bridges with the side chain of D408, which perturbs the H^+^-translocation pathway. Thus, the new inhibitors could prevent either the L to T transition from the inward open to an outward open conformation or deploy the proton motive force necessary for the translocation of substrates by AcrB. Docking and MD simulations in water solution and model phospholipid membrane supported the preference of these EPIs to bind to the L protomer via a cytoplasmic open access channel leading to the TM binding pocket.

### Inhibition of the MFPs

Owing to their functional interaction with the RND transporters, MFPs have been considered a good target for developing inhibitors [12, 149]. In fact, it has been reported that inactivation of these proteins increases antimicrobial susceptibility and prevents the onset of resistance to inhibitors.

**Fig. 9.**
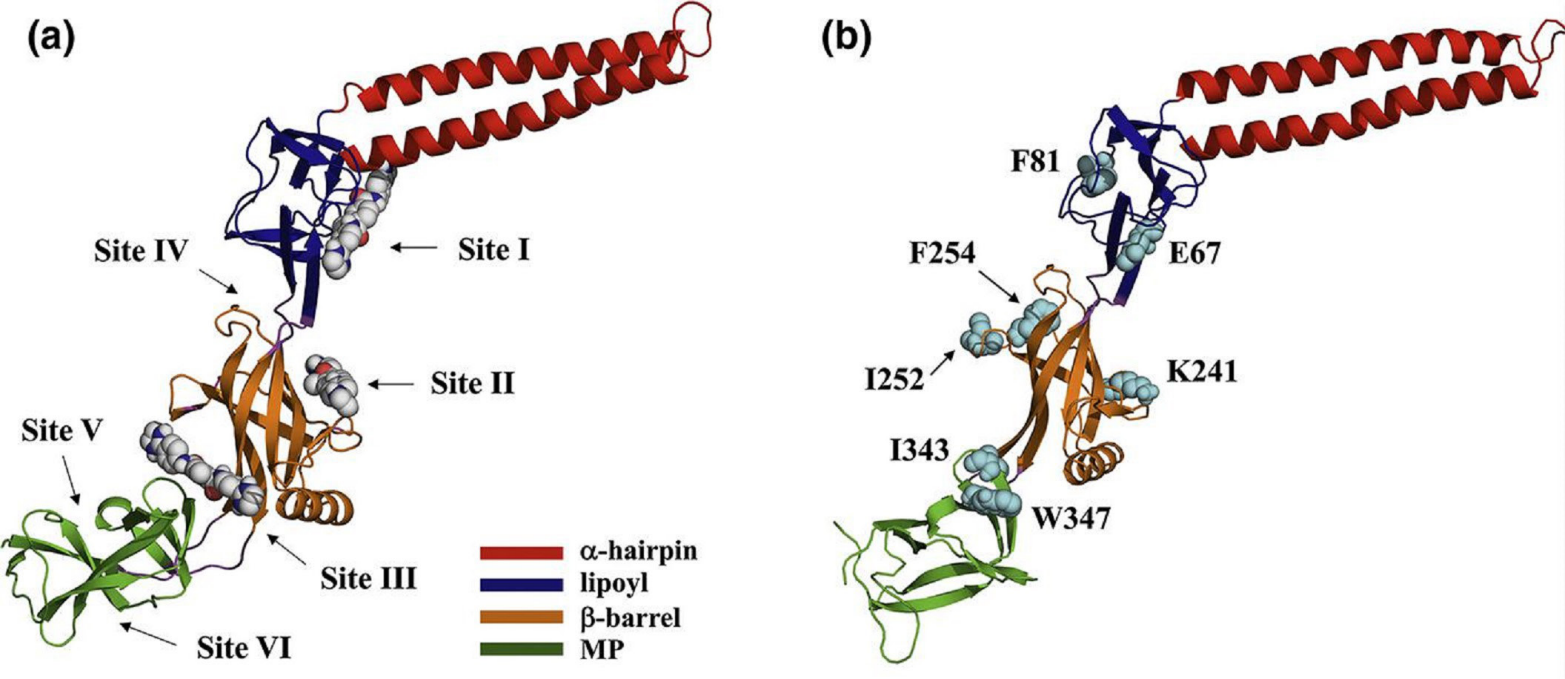
Inhibitor binding sites (a) and major contributing residues (b) in AcrA. Sites I–III are predicted for NSC60339, clorobiocin and novobiocin, and additional sites predicted by FTMap (sites IV–VI). Sites I, II and III are located between the *α*-hairpin and lypoil domains, in the β-barrel domain, and between the β-barrel and MP domain, respectively. Site IV is located between the β-barrel and lypoil domain, and sites V and VI are in the MP domain. Adapted with permission from [152].

Zgurskaya and co-workers complemented experimental work with computer simulations to identify druggable sites onto the MFP AcrA of *E. coli* [150]. They predicted putative binding sites in AcrA and exploited this information in the virtual screening of compounds, which led to the discovery of four new EPIs potentiating the antibacterial activities of novobiocin and erythromycin. These *in silico* results were corroborated by experiments confirming the inhibition of the efflux of fluorescent probes and the potentiation of the activities of several antibiotics in *E. coli* and other Gram-negative bacteria. In the same study, another EPI, named NSC33353 and potentiating the effect of novobiocin and erythromycin in *E. coli *, *A. baumannii *, *K. pneumoniae* and *Enterobacter cloacae *, lacked appreciable intrinsic antibacterial activity of its own in wild-type cells, but it was found to be a substrate for efflux. In study [151], specific chemical modifications led to an analogue of this compound that retain efflux inhibition and gained antibacterial activity in wild-type cells. A different chemical substitution resulted in compounds that lack antibacterial activity but show dual nature, EPI as well as substrates. Ensemble docking calculations performed on both AcrA and AcrB rationalized these findings in terms of the compound’s shifted affinity from AcrA to the AcrB transporter, which makes them better efflux substrates. In a subsequent study [152], the same lab suggested that both the antibiotic clorobiocin and one of these EPIs, NSC60339, bind at a site located between the lypoil and β-barrel domains of AcrA (Fig. 9), as confirmed by tryptophan fluorescence spectroscopy, site-directed mutagenesis and antibiotic susceptibility.

Walker and co-workers [153] performed molecular docking calculations and MD simulations to assist the optimization of compound NSC60339. This work led to the identification of two putative EPIs, SLUPP-225 and SLUPP-417, with improved inhibition activity compared to the parent molecule and potentiation of the activity of novobiocin and erythromycin in *E. coli* cells, as well as enhanced permeation across the outer membrane. Using physico-chemical rules for outer membrane permeability to filter compounds from the ZINC15 database [154], Green *et al.* [155] generated a focused library of compounds to be employed in docking calculations against AcrA. They identified six novel chemical scaffolds that were tested using *in vitro* binding assays and *in vivo* potentiation assays in bacterial strains with controllable permeability barriers and were found to potentiate the activity of erythromycin and novobiocin in *E. coli *, *A. baumannii* and *K. pneumoniae *.

### Inhibition of the OMPs

Several groups employed *in silico* methods with the aim to find EPIs interfering with the transport by and/or the assembly of the OMPs. Verma *et al.* [156] performed high-throughput virtual screening of a large library of biogenic compounds to identify EPIs binding to the outermost component of the RND efflux pump AdeABC in *A. baumannii *. The top complexes formed by putative EPIs with AdeC were further analysed for their binding free energies by MD simulations followed by MM-GBSA calculations. As a result, the EPI ZINC77257599 [(3*R*,4*Z*,6*E*,8*E*)−3-hydroxy-2,2,4-trimethyl-10-oxazol-5-yl-deca-4,6,8-trienamide] was proposed as the lead compound. Seyedhosseini-Ghaheh *et al.* [157] identified crucial conserved residues located at the periplasmic end of the five OMPs, TolC, OprA, OprJ, OprM and OprN. By employing several computational methods, they exploited this information to find putative EPIs acting on OMPs. Subsequent virtual screening campaign led to the identification of 14 ligands, four of which interacted with all the crucial conserved residues. Among these, NPC100251 (Fig. 7) was selected as a potential therapeutic candidate for MDR infections after pharmacokinetic studies.

## Role of inter-protein and membrane-mediated interactions in the functioning of the tripartite pump

Despite the key role of protein–protein and protein–membrane interactions in mediating polyspecificity and transport by RND pumps [5, 10, 12, 14, 15, 158, 159], unveiling the molecular determinants behind their action is extremely challenging. In the last year, several studies, reviewed in this section, shed light on the underlying allosteric mechanisms regulating the efflux of compounds outside Gram-negative bacteria.

**Fig. 10.**
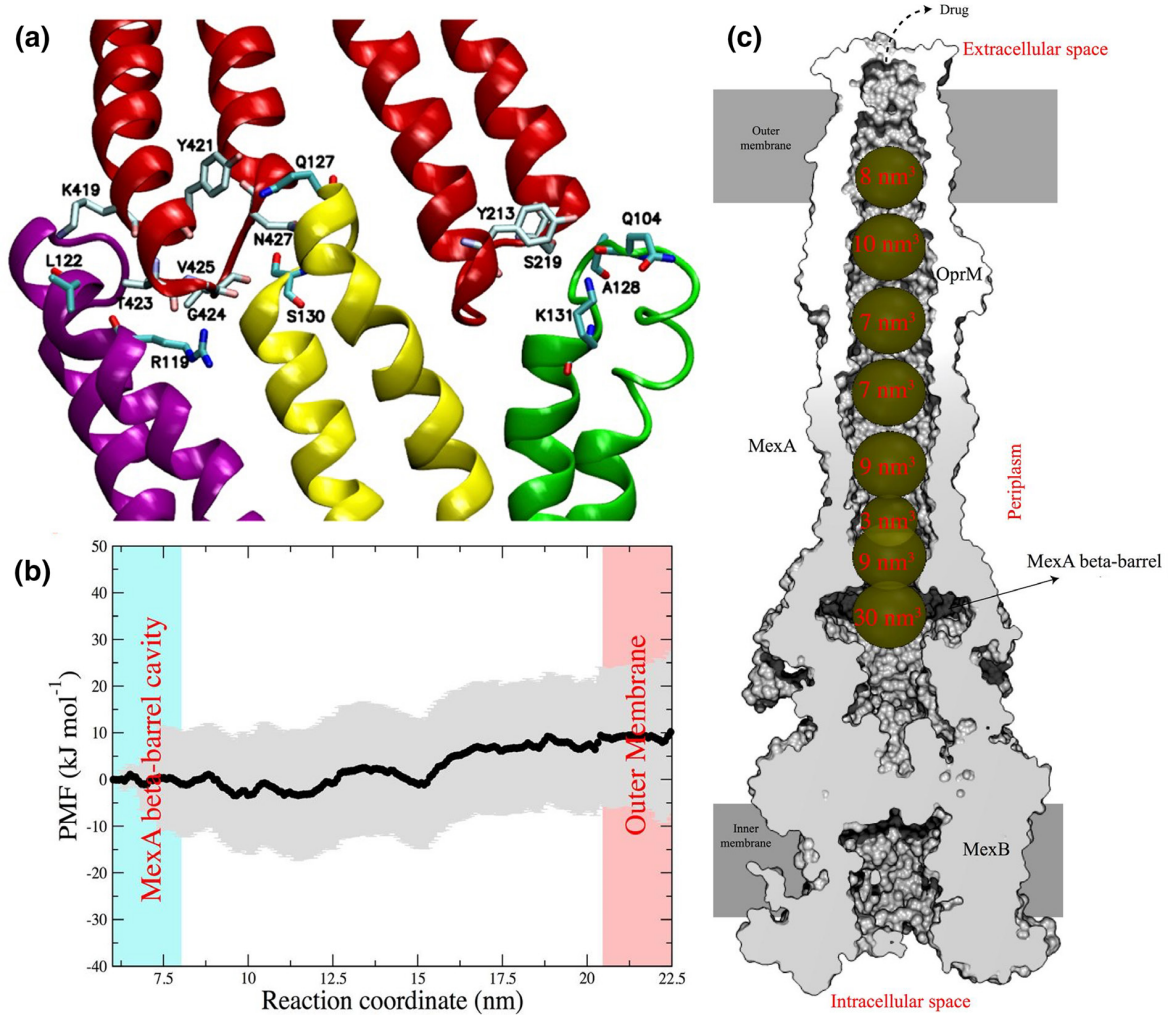
(a) Close-up of the interaction realized between OprM and MexA during MD simulations. A single OprM monomer (red ribbons) was reported to interact with three MexA protomers (green, yellow and purple ribbons)**. (b**) Potential of mean force (PMF) for the translocation of rifampicin through the channel formed by MexA and OprM. The total cumulative energy suggests the process is diffusive as values range within thermal fluctuations**. (c**) Cross-section of the equilibrated fully assembled pump, showing the averaged volume of the conduit along the OprM and MexA complex encountered by the rifampicin drug during its translocation. Adapted with permission from [160].

In 2017, López *et al.* performed the first MD simulations of an intact MexAB-OprM pump in a *P. aeruginosa* bacterial membrane envelope model [160]. By combining all-atom and CG MD simulations with sequence covariation analysis, the authors demonstrated stability of the assembled pump over the microsecond timescale and suggested that the ‘tip–tip’ model with cogwheel-like interactions between the α-hairpins of MexA and OprM is the most likely structure (Fig. 10a), as confirmed in recent cryo-electron microscopy (cryo-EM) structures of MexAB-OprM [24, 161]. Moreover, the comparison between the simulations of OprM in its closed state in the absence and in the presence of MexA highlighted the importance of the MFP for opening of the OMP. Finally, CG MD simulations of the full system allowed capture of the spontaneous translocation of water molecules and rifampicin (Fig. 10b, c).

Two years later, in an effort to shed some light on the role of AcrA in the assembly of AcrAB-TolC, Hazel *et al.* explored its conformational dynamics by combining mutagenesis and functional experiments with MD-based free-energy calculations [162]. The estimated 3D PMF of AcrA displayed two main conformational basins representing assembly competent and incompetent states [163].

Site-directed mutagenesis experiments, introduced at an interdomain interface to shift the dynamic equilibrium in favour of the incompetent one, disrupted pump assembly and function and resensitized *E. coli* to novobiocin and erythromycin antibiotics. This demonstrated the importance of AcrA conformational flexibility for the assembly of AcrAB-TolC and confirmed that modulation of its dynamics through pharmacological intervention is a possible approach to develop new antibiotics.

**Fig. 11.**
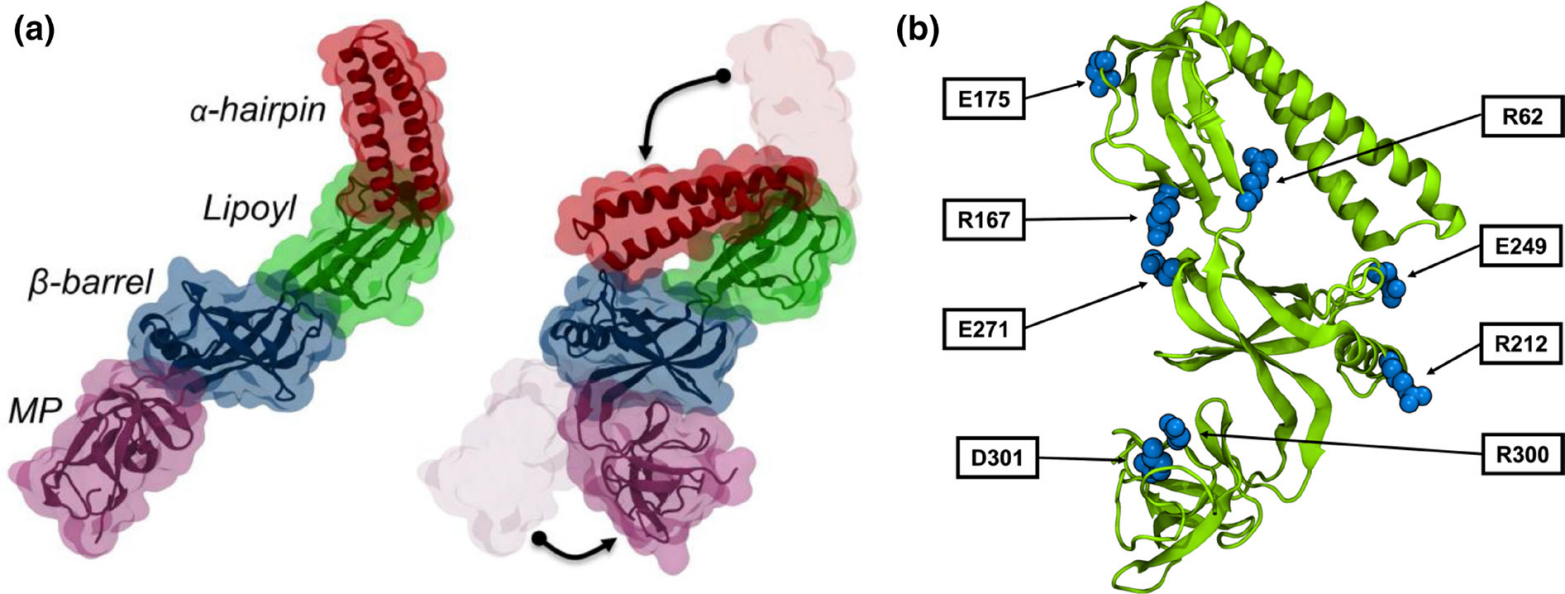
(a) Main conformational changes occurring in the G72S mutated form of MexA after ∼1.5 μs in two independent MD simulations. The X-ray crystal structure elongated shape (left) evolves into a more folded conformation (right), in which the α-hairpin and the MP domains collapse onto the β-barrel domain due to rotation of the peripheral domains (shown by black arrows)**. (b**) MexA_G72S_ representative conformation of the closed state. Pairs of amino acids contributing to stabilization of the complex for more than 10 kcal mol^–1^ are highlighted by skyblue van der Waals spheres. Adapted with permission from [164].

More recently, Cacciotto *et al.* [164] performed computational studies aiming to rationalize the reduced MexAB-OprM functionality in *P. aeruginosa* due to the G72S substitution in MexA [165]. The authors found that: (i) wild-type MexA dimers were stable over multiple microsecond-long atomistic MD simulations and displayed a larger number of native contacts as compared with the G72S variant – these results support a MexA dimer as the functional unit required for the formation of the hexameric assembly found in MexAB-OprM cryo-EM structures [24, 161]; and (ii) the G72S mutation dramatically affected the conformational stability of MexA and resulted in a folding of its peripheral domains on the central regions, leading to a compact structure (Fig. 11a) displaying new intramolecular H-bonds between residues essential for dimerization in the wild-type protein (Fig. 11b). The authors thus concluded that this largely altered equilibrium structure is incompatible with dimerization, explaining the dysfunctional phenotype observed in experiments.

Webber *et al.* investigated the possible allosteric pathways of conformational changes transmitted from the IMP to the MFP and to the OMP in AcrAB-TolC [166]. Long-distance allosteric communication was assessed through a systematic comparison of resting and transport states in terms of distance matrices complemented with evolutionary coupling data and buried surface area estimations. Based on these analyses, the authors hypothesized that the conformational changes causing the opening of TolC are a consequence of substrate binding to AcrB. Moreover, they indicated that the ‘assembled open’ active state has lower energy than the resting (substrate free) state, suggesting the need for energy input (arising from the flux of protons) to bring the efflux pump back to this conformation after the efflux of a substrate. This allosteric model could thus provide hints on how to trap the pump activity in energy minima.

Khalid and co-workers reported in 2017 CG MD simulations of models of the *E. coli* cell envelope incorporating both inner and outer membranes with various embedded native membrane proteins, including the multidrug efflux pump AcrBZ-TolC [167]. Among the interesting outcomes of this study, they noted that LPS molecules in the outer membrane upper leaflet diffuse with a concerted movement and in the same direction of TolC proteins.

In addition, they revealed protein-induced lipid sorting, whereby cardiolipin was significantly enriched within the vicinity of the AcrB–AcrZ complex. The regulatory protein AcrZ is a small protein interacting directly with AcrB [5, 21] and influencing its ability to bind compounds. For instance, Hobbs *et al.* showed that an AcrZ knock-out strain of *E. coli* become sensitive to many of the antibiotics transported by AcrAB-TolC [168]. Du *et al.* exploited their cryo-EM structures of *E. coli* AcrB without and with AcrZ to perform all-atom and CG MD simulations in model IMs containing mixtures of phosphatidylethanolamine and phosphatidylglycerol phospholipids and the modulator cardiolipin [169]. Simulations indicate that conformational changes are induced in the entry channels, gating loops and binding pocket morphology as a result of AcrZ binding. Furthermore, cardiolipin and palmitoyl phosphatidylglycerol were enriched around both proteins, although a slightly higher degree of enrichment was observed around AcrBZ compared with AcrB. The hydrophobic side chains of AcrB were predicted to interact with the lipids’ acyl groups in different ways in the L, T and O protomers, pointing to a role for them in mediating the communication of conformational signals between the protein monomers [159]. Based on experimental and simulation results, the authors suggested that AcrZ and the lipids cooperate to allosterically modulate AcrB activity, through a mechanism that can be shared with other membrane proteins.

**Fig. 12.**
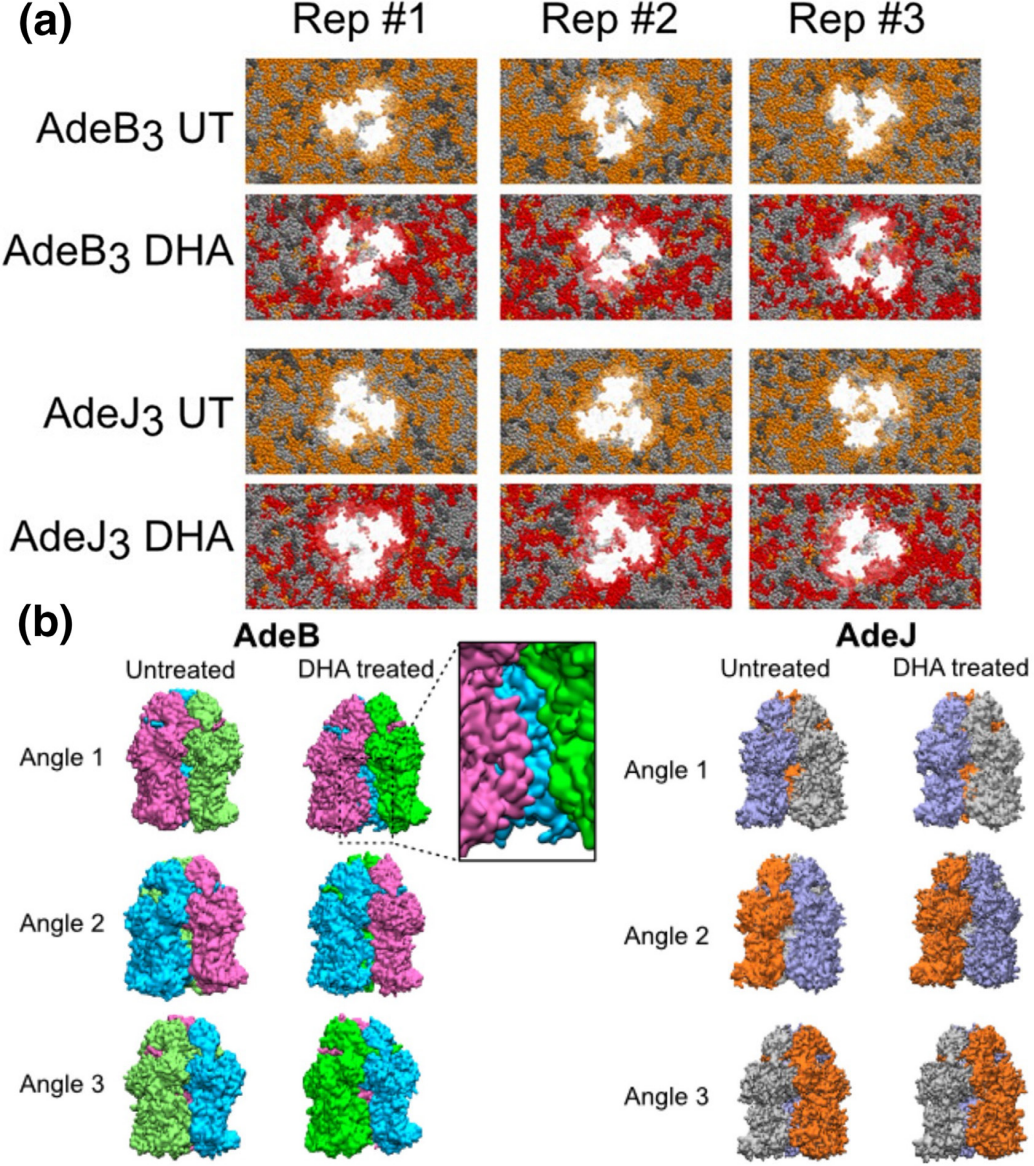
(a) Snapshots of the membrane in the CG MD simulations of AdeB and AdeJ trimers (not shown) embedded in the untreated and DHA-treated membrane performed in triplicate (Rep #1, Rep #2 and Rep #3). Saturated lipids are shown in dark grey, monounsaturated lipids in light grey, diunsaturated lipids in orange and PUFA-containing lipids in red**. (b**) Representative snapshots of AdeB and AdeJ trimer conformations from the MD simulations in (a). Each protomer is coloured differently to aid visualization of protomer interactions from each angle. Adapted with permission from [170].

Zang *et al.* investigated the link between incorporation of host-derived polyunsaturated fatty acids (PUFAs) and increased antibiotic susceptibility in *A. baumannii* [170]. CG MD simulations of AdeB and AdeJ embedded in realistic IM models with and without the ω−3 PUFA docosahexaenoic acid (DHA) highlighted the clustering of unsaturated lipids around both RND transporters (Fig. 12a). However, the AdeJ trimer conformation was largely unaffected by these changes in the local phospholipid environment, while the DHA-enriched membrane produced a specific morphological disruption in AdeB. This resulted in a loss of the protein–protein interface between the TM domains of two adjacent protomers, suggesting a mechanism for the disruption of functional cycling (Fig. 12b).

## Concluding remarks and perspectives

In the last few decades, *in silico* studies have provided valuable clues to several aspects of RND efflux pumps. These include insights into the molecular determinants for their polyspecificity, the mechanisms for substrate transport and those behind inhibition, the assembly of tripartite assembly and the relevance of lipid–protein interactions. These studies have not only supported and rationalized experimental findings, but have also provided original explanations and inspired new research. In the following, we summarize a few methodological developments that could increase the impact of *in silico* investigations on RND efflux pumps and suggest possible future directions for computational studies.

First, while all-atom MD simulations have yielded numerous insights into the molecular aspects of the components of RND machineries, in bacteria these pumps perform their function within an extremely complex environment constituted by the real membrane. The interactions between different components of the tripartite system, and of these proteins with other membrane and periplasmic proteins and with the molecules within the inner and outer membranes could have synergic effects modulating the efflux of compounds outside the cell [171]. It has also been proposed that the wide promiscuity of RND transporters is in part due to these interactions occurring in crowded multi-component systems [166], given that even a small alteration of the network between the transporter and the neighbour proteins falls back on the activity of the transporter itself. These communication/synergic effects make the systems more dynamic, allowing the transporters to adopt a wide range of conformations that probably lead to the binding of a wide variety of ligands.

To provide an example of how these interactions can be crucial, one can think about the still unclear allosteric conformational regulation occurring between the MFP and IMP proteins along the functional cycling of the pump [12], or to the controversy between studies showing that both proton-motive force and substrate binding are necessary to induce a conformational transition from resting (symmetric) to active (asymmetric) states in the IMP [172], and EM-resolved complexes suggesting that this conformational change can occur even without energy supply [173]. To add complexity, the recently discovered new conformation of the IMP associated with an inactive state occurring in the absence of interaction with the MFPs suggests that substrate binding alone might not be crucial to activate the pump [24]. These results raise the issue of how substrate and MFP control conformational transitions and cycle directionality? It is thus clear that the role of auxiliary variables (partner proteins and membrane composition) is crucial in shaping the conformational dynamics of efflux pump components and their remote coupling. A deeper understanding of this synergistic behaviour will aid in the system’s effective and selective targeting. In this scenario, CG descriptions represent a promising tool to bring investigations forward enough to address systems and processes on larger time and/or length scales [174–178]. The increased accuracy of new CG models, coupled with multiscale algorithms and with the potential of exascale computing, could possibly address the full operational cycle of a typical RND efflux pump by means of computer simulations. The concomitant shift from simulations employing simplified model membranes to multicomponent and more realistic ones is critical for understanding the effect of protein–lipid interactions, and the role of the LPS and of the peptidoglycan among other determinants of efflux. All of them are crucial to accurately investigate the machineries in action within the crowded and complex environment of real cells [179]. In addition, simulations of crowded bacterial membranes, comprising additional periplasmic constituents in interaction with efflux pumps, should allow us to accurately mimic *in vivo* counterparts. The importance of such computational studies is reinforced by the limitations of experiments and theoretical models in detecting and modelling the intrinsic complexity of real membranes [175]. Summarizing, future research incorporating chemical details of the local environment will undoubtedly predict more realistic interactions and highlight possibly undetected mechanistic pathways.

Although the polyspecificity of the efflux pumps appears to hamper the development of new effective antimicrobials, this phenomenon could also represent an opportunity in drug discovery, as in the case of repurposing approaches, that could benefit from the ability of the transporters to bind multiple ligands, including already approved drugs [31]. Also in this case, computer simulations can be crucial to address the molecular mechanisms behind possible interactions established between these drugs and RND pump components. Along the same lines of thought, quantum-based simulations, or equivalent approaches modelling reactions and proton transfer, can be of help to shed light on the long-standing dispute regarding the ionization of key residues lining the proton-relay network located in the TM region of the RND transporters [173, 180], which in turn will determine the proton per cycle stoichiometry. Furthermore, coupling these techniques to classic all-atom MD simulations could in principle allow us to investigate how functional movements in the pore domain of these transporters are linked to changes in the protonation states of key residues in the TM region.

Although the idea of using EPIs to improve the efficacy of existing antibiotics holds a great deal of promise, pursuing non-toxic, safe and efficient molecules having such activity has faced so far many issues, and no EPI has been approved to date for treatment in MDR bacteria [181]. A major challenge is related to the broad-spectrum targeting of efflux pumps, as bacteria can simultaneously express multiple different pumps, and as a result targeting just one may not be effective. Therefore, it is highly desirable to identify a common trait (active site, interaction between pump components, energy fuelling, etc.) where several pumps may be targeted [182]. In particular, while the majority of current efforts have been spent to directly target the transport system, new directions are focusing on drugs able to interfere with the assembly of the RND pump and with the transduction of electrochemical into mechanical energy. This quest for more potent EPIs is still being pursued in a combined effort employing cutting-edge computational approaches and collaborations involving scientists with different expertise. Hopefully, more robust platforms, experimental frameworks and advanced techniques such as artificial intelligence and machine/deep learning will make a mark in improving EPI efficacy, safety and spectrum of activity.

Concerning the role of machine learning methods, several studies have already reported their use as a tool to unveil the relationship between the molecular properties of compounds and their propensity to be a substrate and/or inhibitor of efflux systems together with their ability to cross bacterial membranes. The identification of quantitative relationship between structure and properties/activity (QSPR/QSAR) of (candidate) drugs is very useful to filter out thousands of inactive compounds from virtual databases. Usually, machine (deep) learning algorithms are fed with a set of molecular descriptors generated *a priori* from the chemical formula and/or the three-dimensional structure. Among the descriptors mostly used to build QSAR models are various types of topological fingerprints, such as the Extended-Connectivity Fingerprints (also known as Morgan Fingerprints). Graph convolutional networks (GCNs) represent an interesting method to extract several parameters (descriptors) from molecular structures. A paradigmatic example of application of these methodologies in the context of antimicrobials led to the identification of the compound halicin (initially developed for the treatment of diabetes) as a broad-spectrum antibiotic, showing activity against drug-resistant strains of different bacteria [183]. Along the same line, machine learning methods could be used to provide insights into the ADMET (Absorption, Distribution, Metabolism, Excretion and Toxicity) profiles as well as into the most efficient combination therapies, greatly reducing the possibility of developing drug resistance with an increase in efficacy.

To summarize, tripartite efflux pumps underpin several different mechanisms of resistance, virulence and biofilm formation. As such, they are crucial players of antimicrobial resistance, and an important challenge in the development of antimicrobial compounds concerns the identification of active drugs avoiding efflux systems, and/or inhibitors of the latter systems. In the recent years, significant progresses have been made in understanding the physicochemical properties making a drug susceptible to being transported by efflux systems and the specific regions of these machineries involved in transport. However, due to the extreme complexity of these proteins and despite its importance for the design of more effective drugs, a complete understanding of the mechanisms underpinning substrate efflux is still missing. In this context, computational studies have demonstrated to be of great assistance not only to aid the development of new drugs with specific (desired) properties, but also in understanding their fate with the efflux pump. Thanks to the continuous development of more accurate models, community-based tools for the automation of simulations, and increased computing power, we are now able to simulate these systems on a larger time scale and in more realistic and complex environments, enabling researchers to capture key structural and dynamic aspects of these beautiful and so daunting machineries.

## Footnotes

In short, free energies *G* are given by the sum of the interaction energy *E*, the polar and non polar contributions to solvation energy (respectively obtained by means of the generalized Born theory and from the solvent accessible surface area), and the *TS* term associated with entropy (generally neglected). The difference between *G* values calculated for the complex, the protein, and the ligand is the binding free energy D*G_b_*.
